# Model-based orbital-scale precipitation δ^18^O variations and distinct mechanisms in Asian monsoon and arid regions

**DOI:** 10.1093/nsr/nwac182

**Published:** 2022-08-30

**Authors:** Xiaodong Liu, Xiaoxun Xie, Zhengtang Guo, Zhi-Yong Yin, Guangshan Chen

**Affiliations:** State Key Laboratory of Loess and Quaternary Geology, Institute of Earth Environment, Chinese Academy of Sciences, Xi’an 710061, China; University of Chinese Academy of Sciences, Beijing 100049, China; State Key Laboratory of Loess and Quaternary Geology, Institute of Earth Environment, Chinese Academy of Sciences, Xi’an 710061, China; Institute of Geology and Geophysics, Chinese Academy of Sciences, Beijing 100029, China; University of Chinese Academy of Sciences, Beijing 100049, China; Department of Environmental & Ocean Sciences, University of San Diego, San Diego, CA 92110, USA; State Key Laboratory of Loess and Quaternary Geology, Institute of Earth Environment, Chinese Academy of Sciences, Xi’an 710061, China

**Keywords:** orbital scale, precipitation δ^18^O, transient simulation, Asian monsoon, arid region

## Abstract

The past Asian precipitation δ^18^O (δ^18^O_p_) records from stalagmites and other deposits have shown significant orbital-scale variations, but their climatic implications and regional differences are still not fully understood. This study, as the first attempt of a 300-kyr transient stable isotope-enabled simulation, investigated the characteristics and mechanisms of the orbital-scale δ^18^O_p_ variations in three representative regions of Asia: arid Central Asia (CA), monsoonal South Asia (SA) and monsoonal East Asia (EA). The modelling results showed that the variations in the CA, SA and EA annual δ^18^O_p_ exhibited significant but asynchronous 23-kyr precession cycles. Further analyses revealed that although the precession-induced insolation variation was the ultimate cause of the δ^18^O_p_ variation in all three regions, the dominant mechanisms and the involved physical processes were distinct among them. For the CA region, the rainy-season (November–March) temperature effect and water vapour transport by the westerly circulation were identified as the key precession-scale processes linking the October–February boreal mid-latitude insolation to the rainy-season or annual δ^18^O_p_. In the SA region, the rainy-season (June–September) precipitation amount effect and upstream depletion of the monsoonal water vapour δ^18^O served as the main mechanisms linking the rainy-season or annual δ^18^O_p_ to the April–July insolation variation at the precession scale. For the EA region, however, the precession-scale annual δ^18^O_p_ was mainly controlled by the late-monsoon (August–September) and pre-monsoon (April–May) water vapour transport patterns, which were driven by the July–August insolation and the global ice volume, respectively. These results suggest that the climatic implications of the orbital-scale Asia δ^18^O_p_ variations are sensitive to their geographic locations as determined by the combined effects of insolation and regional circulation patterns associated with the respective rainy seasons. This study provides new insights into understanding the regional differences and formation mechanisms of the Asian orbital-scale δ^18^O_p_ variations.

## Teaser

A 300,000-year transient isotope-enabled simulation revealed the regional differences and causes of the orbital-scale precipitation isotope variations in arid Central Asia, monsoonal South Asia and East Asia.

## INTRODUCTION

As a natural tracer, the precipitation oxygen stable isotope O^18^ presented as the ratio δ^18^O (=O^18^/O^16^) has long been regarded as a significant indicator of water-cycle and paleoclimate change [1,2]. Precipitation δ^18^O (abbreviated as δ^18^O_p_ below) is often affected by local meteorological factors, such as precipitation and/or temperature, usually having a negative (positive) correlation with the local precipitation (temperature), which is named as the precipitation amount (temperature) effect [1,3]. Generally speaking, in evaporation, the lighter water molecules (those with O^16^) have a greater tendency to break the molecular bond at the surface and become water vapour [1,4], leading to the enrichment of the heavier water left behind in the water body. Inversely, in condensation, the heavier water vapour molecules (H_2_O^18^) have a greater tendency to change to liquid water, so that the O^18^ content in the water vapour is depleted [1]. In the meantime, the isotope fractionation processes are temperature-dependent. With colder temperatures, the depletion of the heavier water vapour molecules is enhanced [1]. The factors controlling local δ^18^O_p_ also include various large-scale atmospheric processes affecting water vapour oxygen isotope fractionation, such as the changes in the locations of moisture sources or transport pathways, as well as convection, condensation and evaporation in the water vapour source regions and transportation processes [2]. The isotopic records of various sedimentary deposits are often used to reflect past climate changes, in which the cave stalagmite δ^18^O (abbreviated as δ^18^O_c_ below) can inherit the δ^18^O_p_ and thus is closely related to the precipitation or water vapour characteristics, in combination with the advantages of high-resolution and accurate dating [5]. Therefore, δ^18^O_c_ records have been widely used in the field of Asian paleoclimate studies (e.g. [6–8]).

A large number of previous studies on δ^18^O_c_ records of the late Quaternary show that the orbital-scale signals in different locations in the Asian monsoon and arid regions have certain common characteristics [9]. For example, in East Asia (EA) [7,8], South Asia (SA) [10] and Central Asia (CA) [11,12], the δ^18^O_c_ variations are dominated by the quasi-23-kyr (kiloyears, the same below) precession cycle at the orbital scale, suggesting that the precession forcing might have a common controlling effect on the Asian δ^18^O_c_ records, which may reflect the overall intensity of the Asian summer monsoon [13]. However, because the precipitation changes in different regions of Asia are asynchronous, the δ^18^O_c_ records may not directly represent the local precipitation variation, but reflect the changes in atmospheric circulation patterns or water vapour sources [14,15]. Additionally, the EA δ^18^O_c_ records tended to vary asynchronously with the Arabian Sea sedimentary records associated with the Indian monsoon, although the latter also has a strong precession signature [16]. On the other hand, the most significant cyclic pattern recorded by the Chinese loess is the 100-kyr signal (the glacial–interglacial cycle) in the late Pleistocene [17], indicating an important impact of the global ice volume (GIV) on the EA monsoon. Therefore, the climatic implications and impact mechanisms of the Asian δ^18^O_c_ are not entirely clear at this point [18–20].

In recent years, with the development of climate models with the embedded water isotope cycle processes [2,21], several simulation studies have been completed to explore the mechanisms of Asian δ^18^O_p_ variations and the climatic implications of the relevant δ^18^O_c_. Some modelling studies (e.g. [22,23]) show that the change of δ^18^O_p_ in EA depends on the upstream rainout (depletion) process or the upstream effect, which is used to explain the Chinese δ^18^O_c_ records being controlled by precipitation in the upstream Indian Subcontinent. In the meantime, other modelling studies [24,25] show that the variations of the EA δ^18^O_p_ or Chinese δ^18^O_c_ values are the result of the shifts in water vapour sources or atmospheric circulation pathways. Additional simulations suggest that the Asian δ^18^O_c_ records reflect the annual variations of large-scale hydrological processes and circulation regimes [26] or are related to the seasonality of the monsoon precipitation [27]. Although these studies have deepened the knowledge of how δ^18^O_p_ varies in space and time, so far, the controlling factors and regional differences of the δ^18^O_p_ variations at the orbital scales in different regions of Asia are still not fully understood.

With rapid development of high-performance computing technology and application of the acceleration technique of Earth orbital forcing [28], transient simulations through long-term continuous integrations have been increasingly employed in studies on orbital-scale monsoon variations (e.g. [26,29–31]). Xie *et al.* [31] analysed the effects of the orbital-scale insolation forcing and the Northern Hemisphere (NH) high-latitude ice sheets on the EA winter monsoon (EAWM) by using an atmosphere–ocean coupled model in a long-term transient simulation based on the time-varying boundary conditions. Their results show that in the southern EAWM region, its climate variation at the orbital scale is dominated by the 23-kyr cycles in response to the variations of the precession-induced boreal winter insolation, while in the northern EAWM region, the 100-kyr cycle is the dominant pattern due to the strong modulation of the NH ice sheet forcing. Up to now, few existing transient simulations involve the water stable isotope processes or only focus on relatively short time spans, if any (e.g. [32]). In this study, a transient simulation for the past 300 kyr was performed using the same coupled model as in Xie *et al.* [31], in which the water isotope cycle processes are embedded. Our focus on the past 300 kyr is mainly based on three considerations: first, we hope to simulate a sufficiently long time span to capture the characteristics of orbital-scale periodic changes—that is, at least including multiple 100-kyr periods; second, it is necessary to control the integration time in order to save available computational resources; third, there are relatively rich geological records in the simulated geological period to facilitate the reconstruction of boundary conditions and validation of the simulation results. The main goal of the study is to investigate the controlling factors and regional differences of the orbital-scale δ^18^O_p_ variations in three different regions in Asia.

## TRANSIENT SIMULATIONS

The National Center for Atmospheric Research Community Climate System Model version 3 (CCSM3; [33]) was used to conduct the long-term transient simulation in this study for the past 300 kyr [31]. In the transient simulation, various external forcing conditions, such as Earth's orbital forcing, greenhouse gases (GHGs) and global ice sheets, kept varying with time (Supplementary Note 1, abbreviated as Note S1, and so on). Based on this transient simulation, we further conducted another climate simulation for the past 300 kyr using the isotope-enabled CAM3, which is the same as the atmospheric model of CCSM3 but contains the water isotope cycle. The model can describe the processes of isotopic fractionation closely related to surface evaporation and cloud physics [34]. This model has been successfully applied to modelling studies of precipitation isotope changes in the contemporary climate [35] and paleoclimate [22,23]. We used the isotope-enabled CAM3 to simulate the continuous climate change in the past 300 kyr with 100-time orbital acceleration as in Xie *et al.* [31] with the same forcing factors varying with time. However, the time-varying global sea surface temperatures were prescribed according to the output of the transient simulation using CCSM3. In this way, we obtained the changes in the δ^18^O_p_ and atmospheric water vapour δ^18^O (abbreviated as δ^18^O_v_ below) during the past 300 kyr. In addition, the seawater δ^18^O values at the sea surface, which are referred to as the standard mean ocean water at present [36], were obtained at each time step by linearly interpolating from 1.6‰ for 22 ka [37] to 0.5‰ for 0 ka [38], accounting for their changes due to the fluctuation in the sea level during the deglaciation.

**Figure 1.**
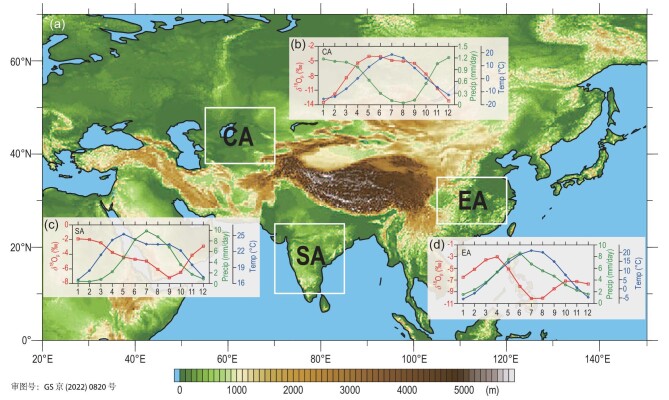
(a) Topography of Asia and the locations of the three study regions: the Central Asian arid region (CA), South Asian monsoon region (SA) and East Asian monsoon region (EA). Diagrams (b), (c) and (d), respectively, show the simulated annual cycles of precipitation (Precip), surface air temperature (Temp) and precipitation oxygen stable isotope ratio (δ^18^O_p_) in the CA, SA and EA regions averaged for the past 300 kyr.

Since this study mainly focuses on the orbital-scale changes of climate and δ^18^O_p_, in the following result analyses, a Gaussian low-pass filter [39] of 5.1 kyr is applied to all simulated climate and δ^18^O_p_ variables in the past 300 kyr to remove the sub-orbital-scale high-frequency climate signals. Regional average climate series of the past 300 kyr (3001 time points at the 100-year resolution) were obtained in three regions in Asia: Central Asian arid region (55–70ºE, 38–50ºN; abbreviated as CA), South Asian monsoon region (70–85ºE, 10–25ºN; as SA) and East Asian monsoon region (105–120ºE, 25–35ºN; as EA) (Fig. 1). When calculating the annual or rainy-season δ^18^O_p_ (δ^18^O_v_) series from the relevant monthly δ^18^O_p_ (δ^18^O_v_) values, the monthly precipitation weighted averages were used (e.g. [40]). In the analyses of the simulation results, we used various time-series analysis and multivariate statistical methods to specify the relationships between the δ^18^O_p_ (δ^18^O_v_) and the forcing factors (see Note S1).

## ANALYSIS OF SIMULATION RESULTS

We first examined the climatological annual cycles of the simulated climate and δ^18^O_p_ variables, and found that they differ significantly in the arid and monsoon regions in Asia. The temperature seasonality in the CA region, averaged for the past 300 kyr (Fig. 1b), shows a single-peak type of annual cycle with a warm summer and cold winter. As an arid climate, its sparse precipitation mainly occurs in winter and spring. However, the annual cycle of the CA δ^18^O_p_ is different from the seasonality of temperature or precipitation, reaching the highest and lowest levels in May and January, respectively. In the SA region, as a typical tropical monsoon climate, its temperature (Fig. 1c) reaches the highest point in May before the onset of the summer monsoon and precipitation is mainly concentrated in summer, while the δ^18^O_p_ values reach the lowest level in September near the end of the summer monsoon. In the EA region, as a subtropical monsoon region, while temperature and precipitation (Fig. 1d) both have approximately synchronous seasonal patterns, the annual cycle of EA δ^18^O_p_ is bimodal with the maxima (minima) in April (August) and October (December), which is significantly different from the annual cycle of the SA δ^18^O_p_ [18]. These distinct characteristics of the annual cycles are generally similar to their modern observations [41–43] in the three regions (Note S2). As a preliminary validation of the modelling results, we also compared the simulated δ^18^O_p_ values for the EA region with Chinese stalagmite records [8]. It shows that their variations are highly in phase with a strong correlation during the past 300 kyr (Note S3).

**Figure 2.**
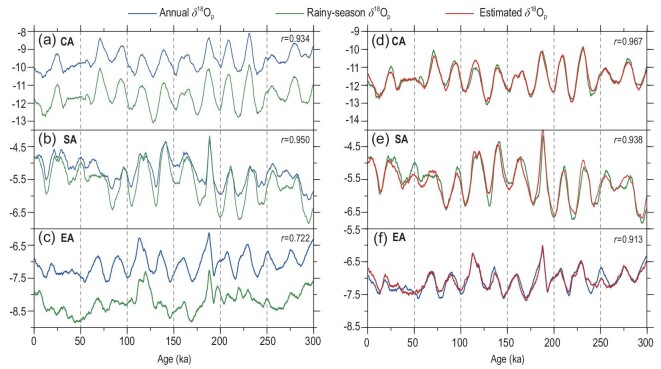
(a) Variations of the annual and rainy-seasonal precipitation δ^18^O_p_ with time in the CA region during the past 300 kyr; (b) same as (a) but for SA; (c) same as (a) but for EA; (d) comparison of the CA rainy-season δ^18^O_p_ with the estimated δ^18^O_p_ using the CA 700-hPa temperature and the whole-troposphere (300-hPa–surface) δ^18^O_v_ as the independent variables; (e) same as (d) but for the SA rainy-season δ^18^O_p_ with the estimated δ^18^O_p_ using the SA precipitation and the water vapour source-region δ^18^O_vs_; (f) same as (d) but for the EA annual δ^18^O_p_ with the estimated δ^18^O_p_ using the EA August–September δ^18^O_p_ and April–May δ^18^O_p_. The *r* values are the correlation coefficients between the two series in each panel.

The rainy seasons occur at different times of the year in these three regions (Fig. 1b–d), specified as November–March in CA, June–September in SA and May–September in EA, respectively (Note S4). By comparing the covariation patterns of the annual δ^18^O_p_ in the past 300 kyr with that of the corresponding rainy seasons of the three regions, it can be concluded that the annual δ^18^O_p_ series in the CA and SA regions have very similar variation patterns to those of the rainy seasons (Fig. 2a and b), with correlation coefficients of 0.934 for the CA and 0.950 for the SA regions, respectively. However, the correlation between the annual and the rainy-season δ^18^O_p_ series is significantly lower in the EA region (*r* = 0.722) (Fig. 2c). This implies that the changes in the CA and SA annual δ^18^O_p_ series are mostly controlled by the changes of the rainy-season δ^18^O_p_, while the EA annual δ^18^O_p_ does not seem to depend as much on the rainy-season δ^18^O_p_. A closer inspection revealed that while the rainy-season δ^18^O_p_ series are more negative than the corresponding annual δ^18^O_p_ series in all three regions, the SA rainy-season δ^18^O_p_ series has peak values very close to the annual values, while the trough values of the rainy-season δ^18^O_p_ are more negative (Fig. 2b). On the other hand, the EA rainy-season series seems to contain more signals of different frequencies than the annual series (Fig. 2c) when compared with the other two regions. These observations imply that the contributions of the rainy-season δ^18^O_p_ to the annual δ^18^O_p_ variations are distinct in different regions.

**Figure 3.**
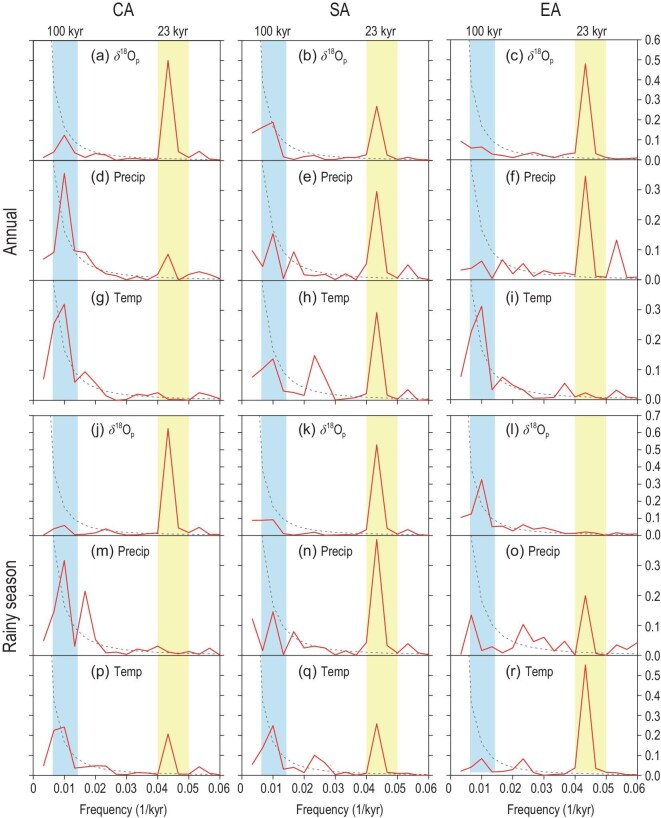
Power spectrum (red lines) analysis results of the standardized annual (a–i) and rainy-season (j–r) δ^18^O_p_, precipitation (Precip) and surface air temperature (Temp) series in the CA (left column), SA (central column) and EA (right column) regions in the past 300 kyr. The black dashed lines indicate the red noise spectrum with 90% confidence level, and the blue and yellow columns indicate the 100-kyr glacial–interglacial cycle band and the 23-kyr precession cycle band, respectively.

Power spectrum analysis of the annual and rainy-season temperature, precipitation and δ^18^O_p_ time series from these three study regions can reveal their orbital-scale variation characteristics in the frequency domain (Fig. 3). It can be seen that the CA (Fig. 3a), SA (Fig. 3b) and EA (Fig. 3c) series all show dominant 23-kyr precession cycles, although weak 100-kyr signals exist in the SA and CA series, which is supported by the Asian stalagmite records [8,44]. Moreover, there are significant 23-kyr cycles in the CA and SA rainy-season δ^18^O_p_ series (Fig. 3j and k) but the EA rainy-season δ^18^O_p_ shows a certain 100-kyr cycle without signals of the 23-kyr cycle (Fig. 3l). Spectral analyses of major climate forcing factors in the late Quaternary (Note S5) show that the GIV and GHG variations are dominated by the 100-kyr cycle, while the annual average insolation by the 41-kyr cycle, and thus they are unlikely to drive the 23-kyr cyclical variation of the CA annual δ^18^O_p_. In the CA and SA regions, there are common precession cycles in the rainy-season (Fig. 3j and k) and annual (Fig. 3a and b) δ^18^O_p_ variations, since the rainy-season series dominate the annual variation (Fig. 2a and b). The rainy-season δ^18^O_p_ variations are ultimately controlled by the precession-induced insolation forcing with prominent 23-kyr cycles (Note S5). However, the analyses in the time domain (Fig. 2c) and frequency domain (Fig. 3c and l) suggest that the EA annual δ^18^O_p_ variation cannot be fully attributed to the rainy-season δ^18^O_p_ variation, as indicated by the mismatch between the 23-kyr cycle in the annual δ^18^O_p_ (Fig. 3c) and the 100-kyr cycle in the rainy-season δ^18^O_p_ (Fig. 3l), which may instead be related to the changes in the GIV or GHGs.

The above analysis shows that the variations in the annual δ^18^O_p_ in the CA, SA and EA regions all indicate significant precession signals at the orbital scale. Although the external forcings of the transient simulation include the GIV and GHG variations dominated by the 100-kyr cycle, the Asian δ^18^O_p_ variations are dominated by the precession cycle. Similar results were also obtained from a previous transient simulation for the NH monsoons using a medium-complexity model [45]. It suggests that the precession forcing plays a leading role in driving the Asian δ^18^O_p_ variations, but the climate-controlling factors and physical mechanisms of the δ^18^O_p_ variations in different regions may be distinct. In the following, we focus on the processes that determine the δ^18^O_p_ variation patterns in each region, in order to clarify the respective variation characteristics and specific controlling factors of the orbital-scale δ^18^O_p_ variation in these typical monsoon and arid regions in Asia since the late Quaternary.

### CA region

Correlation analysis is helpful to infer the relationships between δ^18^O_p_ and precipitation and/or temperature, due to the aforementioned precipitation amount effect and temperature effect [1,40]. In the CA region, there is a significant positive correlation between the δ^18^O_p_ series in the past 300 kyr and annual average temperature (*r* = 0.532), while the positive correlation with precipitation (*r* = 0.294) is contrary to the precipitation amount effect (Table 1). This seems to suggest that the CA annual δ^18^O_p_ variation reflects the temperature effect rather than the precipitation amount effect. However, from the results of the power spectrum analyses, we can see that although the CA annual δ^18^O_p_ series is dominated by the precession cycle (Fig. 3a), annual average temperature does not show the precession cycle (Fig. 3g). Therefore, annual average temperature should not be the direct cause for the variation of the annual δ^18^O_p_. Meanwhile, the correlation between the rainy-season δ^18^O_p_ and temperature is stronger (*r* = 0.685, Table 1) and both δ^18^O_p_ (Fig. 3j) and temperature (Fig. 3p) in the rainy season have significant precession cycles. Therefore, it can be inferred that, similarly to certain continental regions in the NH mid-high latitudes [3,46], there is a strong temperature effect on the variation of δ^18^O_p_ in the rainy season in this region. Since the CA rainy-season δ^18^O_p_ variation dominates the annual δ^18^O_p_ variation, the CA annual δ^18^O_p_ variation also shows a strong signal of the precession cycle. The existing geological evidence shows that the CA water δ^18^O is controlled or affected by temperature [11,47]. The precipitation amount effect on the CA δ^18^O_p_ seems to be weak (if any), although when the temperature's effect is removed, the partial correlations of both annual (*r* = –0.207) and rainy-season δ^18^O_p_ (*r* = –0.234) with precipitation are negative (Table 1).

**Table 1.** Simple and partial correlation coefficients between annual or rainy-seasonal precipitation oxygen stable isotope ratio (δ^18^O_p_), surface air temperature (*T*) and precipitation (*P*) in the Central Asia (CA), South Asia (SA) and East Asia (EA) regions during the past 300 kyr. The rainy seasons in CA, SA and EA are defined as November–March, June–September and May–September, respectively. In partial correlation analysis, *T* and *P* are used as the control variable alternatively.

		Simple correlation			Partial correlation	
		δ^18^O_p_ ∼ *T*	δ^18^O_p_ ∼ *P*	*T* ∼ *P*	δ^18^O_p_ ∼ *T* (*P* as control)	δ^18^O_p_ ∼ *P* (*T* as control)
Annual	CA	0.532	0.294	0.791	0.512	–0.207
	SA	0.048	–0.661	–0.320	–0.230	**–**0.682
	EA	0.372	0.388	0.503	0.222	0.251
Rainy season	CA	0.685	0.251	0.570	0.682	–0.234
	SA	–0.503	–0.797	0.070	–0.742	**–**0.884
	EA	0.035	0.037	–0.003	0.035	0.037

Considering that the CA region is located in the interior of the largest land mass on Earth, the variation of δ^18^O_p_ is not only affected by the surface temperature but also controlled by the upper air temperature [48,49] as the water vapour here has gone through multiple cycles of evaporation and condensation. According to the results of principal component analysis (PCA) of the CA January–December temperature series at various isobaric levels below 300 hPa and the surface (Note S6), there is a consistent variation pattern of the temperature in the winter months (October–February), with an antiphase temperature variation pattern in the summer months, which result from the temperature responses to the precession-induced winter–summer opposite insolation variations as shown in an earlier study [30]. From the correlations between the CA rainy-season δ^18^O_p_, tropospheric temperature and δ^18^O_v_ (Note S7), the δ^18^O_p_ series has strong positive correlations with the tropospheric temperatures and the highest correlation is with the 700-hPa temperature (*r* = 0.770), which is higher than the correlation with the surface temperature (*r* = 0.685, Table 1). We also find a high positive correlation (*r* = 0.823) between the CA rainy-season δ^18^O_p_ and the δ^18^O_v_ of the whole troposphere (300-hPa–surface, same below), which is independent of the tropospheric temperature (Note S7). Based on these results, we used the 700-hPa temperature and whole-troposphere δ^18^O_v_ as the independent variables to estimate the values of the CA rainy-season δ^18^O_p_ in regression analysis. The estimated and simulated rainy-season δ^18^O_p_ series match each other extremely well, with a correlation coefficient of 0.967 (Fig. 2d). In other words, the 700-hPa temperature and the whole-troposphere δ^18^O_v_ can explain 93.5% of the variance in the CA rainy-season δ^18^O_p_.

Since the effects of orbital forcing are ultimately reflected in the variations of insolation at different latitudes of Earth [50], at the orbital scale, both the upper air temperature and δ^18^O_v_ should be related to the insolation variation. By calculating the correlations between January–December monthly temperature series at 700 hPa in the CA region and the insolation at approximately the same latitudes (45ºN), it is found that the correlation coefficients between the monthly average temperature and the leading 1- to 2-month insolation are usually the highest (Note S8). Thus, the CA rainy-season 700-hPa temperature and the whole-troposphere δ^18^O_v_ related to the δ^18^O_p_ are both strongly associated with the average October–February mid-latitude insolation, with correlation coefficients of 0.619 and 0.877, respectively (Note S8).

**Figure 4.**
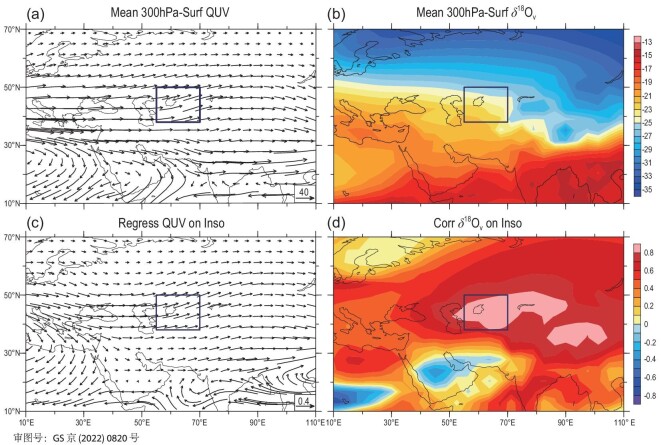
Distribution patterns of the whole-troposphere (a) water vapour flux over Eurasia (units: kg/m/s), (b) water vapour δ^18^O_v_ averaged for the CA rainy season (November–March) in the past 300 kyr, as well as (c) the regression field of the water vapour flux (units: kg/m/s) against the October–February 45ºN insolation and (d) correlation field of the rainy-season δ^18^O_v_ with the insolation. The dark-blue boxes indicate the CA region.

In order to further understand how the temperature and δ^18^O_v_ are modulated by the insolation and what specific physical processes are affecting the δ^18^O_p_, we examined the spatial patterns of whole-troposphere water vapour flux and transport in relation to the 45ºN insolation (Fig. 4). In terms of the climatological average conditions, water vapour in the CA region mainly comes from the transport by the westerly circulation during the rainy season (Fig. 4a). Previous studies have shown that the westerly circulation is the main mechanism transporting water vapour into CA in the late Quaternary [30] or even during the entire Cenozoic [51]. In the Eurasian continent, the CA rainy-season δ^18^O_v_ is generally high in the south and low in the north (Fig. 4b), which reflects the temperature effect to a certain extent [11,47]. However, in the mid-latitude westerly-circulation controlled areas, from the northern Mediterranean, through the Black Sea and Caspian Sea, to the downstream CA near the Aral Sea, the δ^18^O_v_ values are gradually becoming more negative (Fig. 4b), which may reflect the upstream depletion effect influencing the water vapour isotope content. Regression analysis shows that when the insolation in the cold season increases with the precession cycle, the westerly circulation enhances and transports more water vapour with more positive δ^18^O_v_ upstream to the downstream areas (Fig. 4c), resulting in more positive (or less negative) δ^18^O_v_ in the CA region. On the other hand, the insolation enhancement also causes the CA δ^18^O_v_ to shift toward more positive (or less negative) through the temperature effect on fractionation. The combined action of these two results in a highly positive correlation between the CA δ^18^O_v_ and the insolation (Fig. 4d and Note S8). It is thus clear that the precession-induced mid-latitude insolation variation ultimately controls the CA δ^18^O_p_ at the orbital scale by modulating the tropospheric temperature and δ^18^O_v_ during the rainy season.

### SA region

Although there are prominent precession signals in both annual precipitation (Fig. 3e) and annual mean temperature (Fig. 3h), there is no correlation between the annual δ^18^O_p_ and annual mean temperature (*r* = 0.048, Table 1), while a significant negative correlation exists between the annual δ^18^O_p_ and annual precipitation (*r* = –0.661). Therefore, it is preliminarily inferred that the variation of the SA annual δ^18^O_p_ mainly reflects the precipitation amount effect. Since the SA precipitation is concentrated during the rainy season, further analysis of the relationship between the rainy-season δ^18^O_p_ and precipitation shows a strong negative correlation (*r* = –0.797). However, there is also a moderate negative correlation between δ^18^O_p_ and rainy-season temperature (*r* = –0.503), which is contrary to the temperature effect. Although the SA rainy-season δ^18^O_p_ (Fig. 3k), temperature (Fig. 3q) and precipitation (Fig. 3n) all show significant precession cycles, the peak value of the standardized power spectrum of temperature in the precession band (∼23 kyr) is significantly lower than that of precipitation. When the influence of temperature is excluded using partial correlation analysis (Table 1), the negative relationship between the rainy-season δ^18^O_p_ and precipitation becomes even stronger (partial *r* = –0.884). The PCA of the SA January–December δ^18^O_p_ series also shows a consistent variation pattern of monthly δ^18^O_p_ during June–September, identified as the SA rainy or monsoon season (Note S9). Therefore, it can be concluded that the SA annual δ^18^O_p_ may mainly depend on the precipitation amount effect during the rainy season, which is also supported by existing research results [52–54].

**Figure 5.**
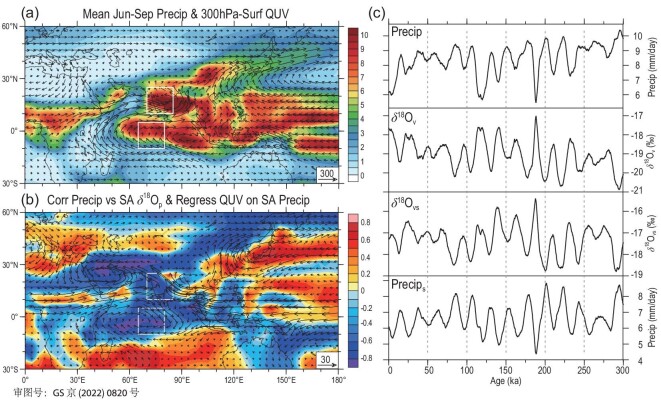
(a) Precipitation (shaded, units: mm/d) and whole-troposphere water vapour flux (arrows, units: kg/m/s) fields averaged for the SA rainy season (June–September); (b) correlation field of the rainy-season precipitation with the SA δ^18^O_p_ series (shaded) and the regression field of the water vapour flux (arrows, units: kg/m/s) against the SA rainy-season precipitation series; (c) comparisons between the SA rainy-season (June–September) precipitation (Precip), whole-troposphere δ^18^O_v_ and the source-region δ^18^O_v_(δ^18^O_vs_) and precipitation (Precip_s_) series. In (a) and (b), the white boxes in the NH indicate the SA region, and those in the SH represent the water vapour source region for SA precipitation in this study.

For the past 300 kyr, the Indian Subcontinent has been one of the regions with the highest precipitation amount in the world during June–September, the rainy season of the SA region (Fig. 5a). Its water vapour mainly originates from the southern Indian Ocean (Fig. 5a). Correlation analysis between the SA rainy-season δ^18^O_p_ series and the simultaneous precipitation field (Fig. 5b) revealed that the SA δ^18^O_p_ series has strong negative correlations not only with the local precipitation, but also with the precipitation in the central tropical Indian Ocean extending to the Southern Hemisphere (SH). The increase in precipitation in the SA region is accompanied by the enhancement of water vapour transport from the southwest (Fig. 5b). This means that the variation of the SA δ^18^O_p_ is not only controlled by the local precipitation effect, but also closely related to the precipitation in the southern tropical Indian Ocean, or the upstream depletion effect. For simplicity, a region of the central tropical Indian Ocean (65º–80ºE, 10ºS–5ºN) is selected as the upstream water vapour source region of SA precipitation (Fig. 5a and b). Moreover, the source region is very stable, as judged from similar correlation patterns of the δ^18^O_v_ field with the SA δ^18^O_p_ series in the glacial and interglacial periods during the past 300 kyr (Note S10).

By comparing the SA rainy-season δ^18^O_p_ series with the local precipitation and water vapour δ^18^O_v_, as well as the source-region whole-troposphere δ^18^O_v_ and precipitation series (Fig. 5c), it can be seen that they all have marked precession cycles with almost synchronous changes. Taking the SA local precipitation and the source-region δ^18^O_v_ in the rainy season as two relatively independent influencing factors, we estimated the SA δ^18^O_p_ series using multiple regression. The correlation coefficient between the estimated and the original simulated δ^18^O_p_ series reaches 0.938 (Fig. 2e). Considering that the correlation between the SA rainy-season precipitation and δ^18^O_p_ is –0.797 (Table 1), explaining 63% of the variation in the SA δ^18^O_p_, the source-region δ^18^O_v_ can explain 73% of the variation, while the combination of the two can account for 88% of the variance of the SA δ^18^O_p_.

**Figure 6.**
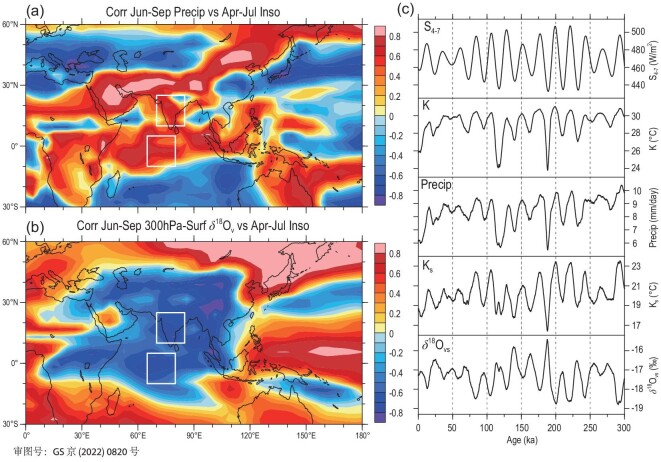
(a) Distribution of correlation coefficients of June–September precipitation in the Asia–Indian Ocean region with the April–July 30ºN insolation series; (b) same as (a) but for the whole-troposphere δ^18^O_v_ with the insolation; (c) temporal variation patterns of the April–July 30ºN insolation (S_4_7_), SA June–September atmospheric stability index (K), SA rainy-season precipitation (Precip), the water vapour source-region K index (K_s_) and whole-troposphere δ^18^O_vs_ during the past 300 kyr. The white boxes are the same as in Fig. 5.

Results of correlation analysis indicate that the SA precipitation series of each month in the rainy season is usually most closely related to the 30ºN insolation leading by 2 months, thus the SA precipitation in the rainy season (June–September) has the strongest positive correlation with the April–July insolation, while the SA δ^18^O_p_ or the source-region δ^18^O_v_ has the strongest negative correlation (Note S11). Similarly, the correlation fields also show that the April–July average 30ºN insolation has strong positive correlations with the June–September precipitation over the vast tropical areas including the SA and the source region (Fig. 6a), but strong negative correlations are found with the June–September whole-troposphere δ^18^O_v_ over most parts of the Asian continent and tropical Indian Ocean (Fig. 6b). This suggests that when the precession forcing causes the insolation to enhance (weaken) for the NH spring and summer, it can result in increases (decreases) in the SA rainy-season precipitation and the depletion (enrichment) of the source-region δ^18^O_v_, eventually leading to the more negative (positive) δ^18^O_p_ values in the SA region through the local precipitation amount effect and upstream depletion effect.

To explain the insolation-induced atmospheric physical processes affecting the SA precipitation and the source-region δ^18^O_v_, we further analysed the atmospheric water vapour and stability variations related to the orbital forcing. The regression analysis results show that when the April–July 30ºN insolation is strengthened, the water vapour transport into the SA from the southwest is enhanced, resulting in increases in the SA rainy-season water vapour content (and precipitation) (Note S12). Meanwhile, the enhanced insolation may affect atmospheric stability and convective activity. The atmospheric instability and convective intensity can be measured using the K index [55], i.e. *K* = (*T*_850_ – *T*_500_)  + *D*_850_ – (*T*_700_ – *D*_700_). The first term (*T*_850_ – *T*_500_) is the air temperature lapse rate between 850 and 500 hPa, and the second term *D*_850_ and the third term (*T*_700_ – *D*_700_) are respectively the dew point temperature at 850 hPa and the 700-hPa dew point depression, reflecting the lower tropospheric humidity. The higher the K index, the stronger the instability and convective activity of the atmosphere. In relation to our simulation results for the past 300 kyr, the enhancement of April–July 30ºN insolation caused by the precession cycle leads to the rise in the K index over the SA region during the rainy season, which is also conducive to the synchronous increase of SA monsoon precipitation (Fig. 6c). At the same time, the enhanced insolation also causes the rise of the K index over the tropical Indian Ocean, resulting in the enhancement of convective precipitation and then the corresponding depletion of the source-region δ^18^O_v_ level (Fig. 6c). As a result, the enhanced insolation at the orbital scale in the NH spring and summer increases the local precipitation and lowers the upstream δ^18^O_v_ by promoting convective activities in both local and water vapour source areas and enhancing the water vapour transport to the SA region, leading to the further depletion of the SA rainy-season δ^18^O_p_. When the precession-induced insolation weakens, the opposite would occur.

### EA region

Unlike the CA and SA regions, the EA annual δ^18^O_p_ is not controlled by the rainy-season temperature or precipitation. From the results of power spectrum analysis, there are significant precession signals in the EA annual δ^18^O_p_ series (Fig. 3c) and annual precipitation (Fig. 3f). However, there is no precession signal in the EA annual mean temperature (Fig. 3i) or rainy-season δ^18^O_p_ (Fig. 3l). Although the annual δ^18^O_p_ is positively correlated with the annual average temperature (*r* = 0.372, Table 1) and precipitation (*r* = 0.388), the rainy-season δ^18^O_p_ is almost irrelevant to the variation of rainy-season temperature (*r* = 0.035) or precipitation (*r* = 0.037). It can be seen that the EA annual δ^18^O_p_ variation at the orbital scale cannot be simply attributed to the variation of temperature or precipitation during the rainy season. Partial correlation analysis does not significantly change the original relationships between the rainy-season δ^18^O_p_ and temperature or precipitation (Table 1).

Based on the PCA results and correlation analysis of the January–December EA δ^18^O_p_ series, August–September and April–May δ^18^O_p_ have been identified as the highest loading contributors to the variation in the EA annual δ^18^O_p_ (Note S13). In fact, if the two series of August–September and April–May δ^18^O_p_ are used to estimate the annual δ^18^O_p_ in the EA region using regression analysis (Fig. 2f), the estimated series is found to be highly consistent with the simulated annual δ^18^O_p_ (*r* = 0.913)—that is, the August–September and April–May δ^18^O_p_ can explain 83.4% of the EA annual δ^18^O_p_ variation. Actually, only August–September δ^18^O_p_ alone would be able to explain 61.9% of the annual δ^18^O_p_ variation. Therefore, the late rainy season is the key period to determine the variation in the EA annual δ^18^O_p_.

**Figure 7.**
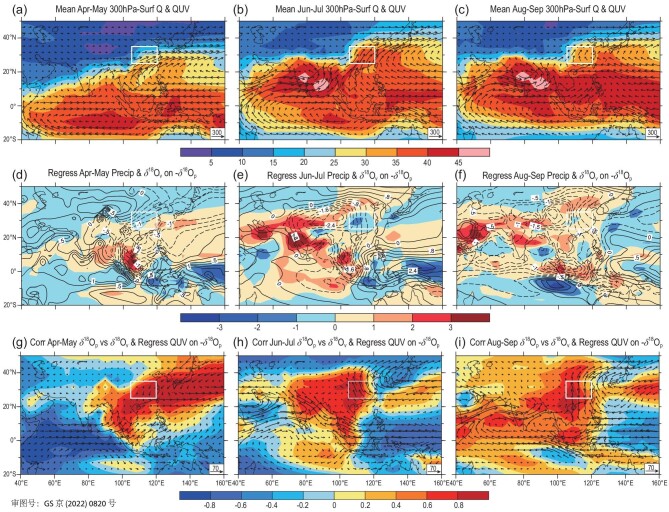
Fields of the whole-troposphere water vapour content (shaded, units: kg/m^2^) and water vapour flux (arrows, units: kg/m/s) averaged for (a) April–May, (b) June–July and (c) August–September; regression fields of the whole-troposphere water vapour δ^18^O_v_ (contour) and precipitation (shaded, units: mm/d) with the –δ^18^O_p_ series of EA for (d) April–May, (e) June–July and (f) August–September, respectively; correlation fields of the δ^18^O_v_ with the EA δ^18^O_p_ (shaded) and regression fields of the whole-troposphere vapour flux (arrows, units: kg/m/s) against the –δ^18^O_p_ series of EA for (g) April–May, (h) June–July and (i) August–September. The white boxes indicate the EA region.

The EA August–September and April–May δ^18^O_p_ series are closely related to the concurrent local mid-lower-troposphere water vapour δ^18^O_v_ as shown in the corresponding correlation analysis (Note S14). The variation in the δ^18^O_v_ over EA mainly depends on the water vapour transport [22] related to the Asian summer monsoon, but the relevant large-scale circulation pattern varies greatly in different stages of the monsoon season [56,57]. For the convenience of discussion in the following, we take three sub-seasonal stages, namely April–May, June–July and August–September, to represent the pre-, early- and late-monsoon stages of the EA region, respectively. Based on the long-term average conditions, the water vapour flux entering the EA region is dominated by the southerly air flow, but mainly affected alternately by the winds from the South China Sea, Arabian Sea–Indian Ocean and western Pacific during the pre-, early- to late-monsoon seasons (Fig. 7a–c).

According to the regression and correlation analyses, for the pre-monsoon season, the depletion of the EA δ^18^O_p_ corresponds to the more negative δ^18^O_v_ values in the vast areas of south-eastern China, Indochina Peninsula, South China Sea and western Pacific (Fig. 7d, represented by the dashed contours). In other words, the EA δ^18^O_p_ is highly positively correlated with the δ^18^O_v_ in these areas (Fig. 7g). However, the depletion of the EA δ^18^O_p_ due to the enhanced northward water vapour transport (Fig. 7g) has little to do with the precipitation variation in most of the Asian monsoon region (Fig. 7d), because the rainy season has not yet begun. As a comparison, there are significant positive correlations between the EA δ^18^O_p_ and the δ^18^O_v_ in most parts of the Asian continent in the early-monsoon season (Fig. 7h), corresponding to the more depleted δ^18^O_v_ values in this general region with the depletion of EA δ^18^O_p_ (Fig. 7e). In the estimated water vapour flux field, the variation of the EA δ^18^O_p_ does not seem to be directly related to the anomalous water vapour transport from the upstream Arabian Sea to SA, but more so to the water vapour transport from the western Pacific/East China Sea (Fig. 7h). Therefore, although the EA water vapour in the early-monsoon season climatologically mainly comes from the south-westerly flow over the Indian Ocean (Fig. 7b), the June–July south-westerly water vapour transport makes little contribution to the orbital-scale variation of the EA annual δ^18^O_p_.

It is worth noting that in the late-monsoon season (Fig. 7f), the depletion of the EA δ^18^O_p_ corresponds to the decreases of δ^18^O_v_ in most parts of the Asian continent and the upstream tropical Indian Ocean, as well as the increases of precipitation near the Arabian Peninsula, Indian Subcontinent and Sumatra Island. Accordingly, the EA δ^18^O_p_ series has highly positive correlations with the variation of δ^18^O_v_ from the upstream South China to the Arabian Sea (Fig. 7i), and is also related to the water vapour transport from the northern Arabian Sea to SA and then into EA (Fig. 7i). In fact, the stronger the water vapour transport from the upstream to the EA region, the more negative the EA δ^18^O_p_ tends to be due to the upstream depletion effect (Fig. 7i). It is thus clear that although the water vapour over the EA region climatologically mainly comes from the South China Sea and the western Pacific during the late-monsoon season (Fig. 7c), the orbital-scale variation of EA δ^18^O_p_ actually depends on the water vapour transport from the Arabian Sea to SA. Several previous simulation [22,23] and observation [43,58] studies have pointed out that the variation of δ^18^O_p_ in EA is largely controlled by the upstream convective activity or rainout process. However, here we want to suggest that such a mechanism plays a role mainly in the late-monsoon season.

**Figure 8.**
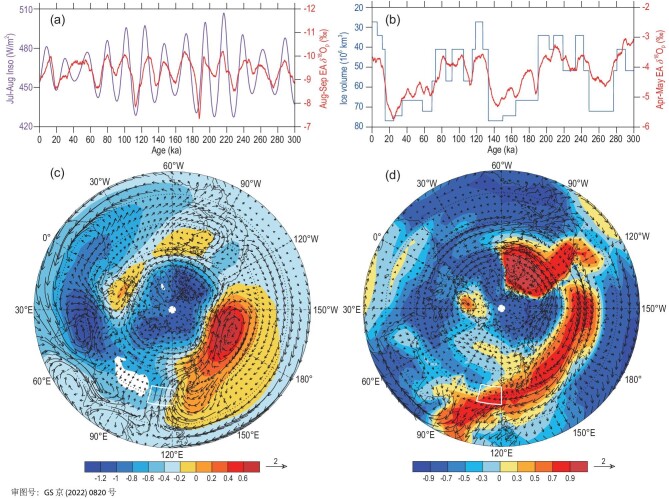
(a) Time series of the EA August–September δ^18^O_p_ and July–August 30ºN insolation series; (b) the EA April–May δ^18^O_p_ series and the GIV series used in the model; (c) the regression NH fields of the August–September average 850-hPa geopotential heights (shaded, units: gpm) and the whole-troposphere water vapour flux (arrows, units: kg/m/s) against the July–August 30ºN insolation; (d) the NH distributions of correlations of the April–May whole-troposphere δ^18^O_v_ with the negative GIV series (shaded) and the regression field of the water vapour flux (arrows, units: kg/m/s) against the negative GIV series. The white boxes in (c) and (d) indicate the EA region, and the white shaded area in (c) shows the Tibetan Plateau.

In terms of the forcing mechanisms, we find that the August–September EA δ^18^O_p_ and water vapour content have strong positive correlations with the July–August insolation, while the April–May δ^18^O_p_ and water vapour content are strongly correlated with the variations of GIV and GHGs (Note S15). The time series of the EA August–September δ^18^O_p_ plotted against the July–August 30°N insolation (Fig. 8a) and the EA April–May δ^18^O_p_ against the GIV (Fig. 8b) show close associations between the δ^18^O_p_ and the respective forcings. The regression analysis suggests that, corresponding to increasing insolation and thus stronger warming over the land mass than that over the ocean (Fig. 8c), the 850-hPa geopotential heights rise over the North Pacific and fall over the Asian continent centred on West Asia, resulting in enhanced water vapour transport from the Arabian Sea and SA to EA due to enhanced low- to mid-latitude pressure gradients across 60ºE–110ºE. This anomalous circulation pattern leads to the reduced EA δ^18^O_p_ through the upstream depletion effect (Fig. 7i). On the other hand, the retreat of the NH ice sheets induces the decline of the geopotential heights in the Arctic region and the rise of geopotential heights in the mid-latitude North Pacific to EA, resulting in the increased water vapour transport from the western Pacific into the EA region (Fig. 8d). This situation favours the EA δ^18^O_p_ to shift towards a more positive level (Fig. 7g). Therefore, the increase (decrease) of the July–August insolation due to the precession cycle combined with the expansion (retreat) of the NH ice sheets is most conducive for the EA δ^18^O_p_ content to be depleted (enrichened). Since the EA annual δ^18^O_p_ mainly depends on the August–September δ^18^O_p_ variation and the contribution by the April–May δ^18^O_p_ is relatively small, we can conclude that the variation of the EA annual δ^18^O_p_ is mostly controlled by the July–August insolation reflecting the effects of the precession cycle, although it is also related to the fluctuation of the GIV (including the GHG concentration) at the precession scale (Note S5).

### Regional comparisons

**Figure 9.**
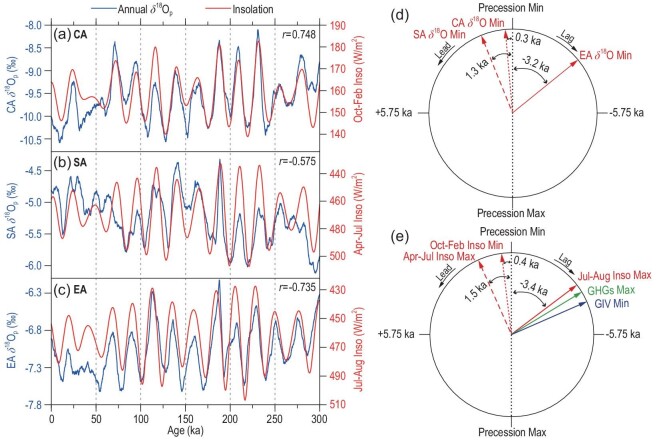
(a) Comparisons of the CA annual δ^18^O_p_ with October–February average 45ºN insolation series; (b) same as (a) but for the SA δ^18^O_p_ with April–July 30ºN insolation; (c) same as (a) but for the EA δ^18^O_p_ with July–August 30ºN insolation; (d) the phase relationships between the precession parameter minimum (Min), the simulated CA, SA and EA annual δ^18^O_p_ minima in the precession band, and (e) the phase relationships between the precession parameter (Min) and the forcing factors including the October–February 45ºN insolation (Inso) minimum, April–July 30ºN insolation maximum, July–August 30ºN insolation maximum, global ice volume (GIV) minimum and concentrations of greenhouse gases (GHGs) maximum (CO_2_ equivalent).

The above analyses show that the simulated annual δ^18^O_p_ variations in the CA, SA and EA regions all have the dominant 23-kyr cycle, but are driven by the precession-induced insolation of different months. In terms of the time domain, the CA, SA and EA annual δ^18^O_p_ show almost completely synchronous changes at the orbital scale with the October–February, April–July and July–August NH insolation, respectively (Fig. 9a–c), although there seems to be the signal of the 100-kyr cycle in the SA region (Fig. 9b). It can be observed more clearly in the frequency domain (Fig. 3b). The cross-spectral analysis reveals the phase relationships of the simulated CA, SA and EA annual δ^18^O_p_ series relative to the precession parameter (Fig. 9d). For the 23-kyr precession cycle, the annual δ^18^O_p_ series of the CA and SA regions lead the minimum of precession parameter (corresponding to the NH June insolation maximum at mid-low latitudes) by ∼0.3 and ∼1.3 kyr, respectively; while the EA annual δ^18^O_p_ series lags the minimum of the precession parameter by ∼3.2 kyr. In other words, the EA annual δ^18^O_p_ lags the SA (CA) by 4.5 kyr (3.5 kyr), while the CA annual δ^18^O_p_ lags the SA annual δ^18^O_p_ by only 1 kyr. This indicates that although the variations of the annual δ^18^O_p_ series in all three regions are dominated by the precession cycle, their phase relations with the precession band are not entirely the same.

Compared with the phases of the various forcing factors at the precession scale (Fig. 9e), we can see the corresponding relationships of the δ^18^O_p_ in these three regions and different factors. The phase of the temperature-affected CA δ^18^O_p_ minimum is very close to that of the October–February minimum insolation, the phase of the precipitation-affected SA δ^18^O_p_ minimum is close to that of the April–July insolation maximum, while the EA δ^18^O_p_ minimum is close to the July–August insolation maximum as well as the GHGs maximum and GIV minimum. These phase relationships confirm that the variations of CA and SA annual δ^18^O_p_ are mainly decided by their rainy-season δ^18^O_p_ and ultimately controlled by the corresponding NH insolation, while the EA δ^18^O_p_ is mainly controlled by the late-monsoon insolation and it is probably also related to the variations of GIV and GHGs. In the precession cycle, there exist fixed phase differences of the insolation variations of different months [50]. Since the CA, SA and EA annual δ^18^O_p_ variations in the precession band are determined by the insolations of different months (Fig. 9), there should also be relatively stable phase differences between the variations of the δ^18^O_p_ in these regions. However, these modelling results remain to be verified with geological records available for different regions in the future.

## CONCLUSIONS AND DISCUSSIONS

In this study, the transient climate simulation spanning the past 300 kyr was carried out using a global atmosphere–ocean coupled model and the corresponding isotope-enabled atmospheric model. Based on the analyses of the simulation results, the characteristics and mechanisms of the orbital-scale variations of δ^18^O_p_ in three representative regions of Asia (the CA, SA and EA region) are investigated and compared. The results show that the orbital-scale variations of the CA, SA and EA δ^18^O_p_ are all dominated by the 23-kyr precession cycle, although weak 100-kyr signals exist in the CA and SA regions. However, the precession-scale δ^18^O_p_ variations are not synchronous, with certain phase differences between these regions of Asia. At the precession scale, the EA annual δ^18^O_p_ lags those of the SA and CA regions, by ∼4.5 and 3.5 kyr, respectively, and the CA annual δ^18^O_p_ is ∼1 kyr behind that of the SA region. Our results indicate that the annual δ^18^O_p_ variations at the precession scale in the three regions are driven by the insolation forcing in different months. Therefore, the inherent phase differences of the insolation in different months in the precession cycle create relatively stable phase relationships between the annual δ^18^O_p_ series of these regions.

Although the δ^18^O_p_ in the three regions studied have significant precession signals at the orbital scale, the physical mechanisms of their formations are distinct. The CA annual δ^18^O_p_ variation mainly depends on the rainy-season (November–March) δ^18^O_p_. The NH mid-latitude insolation in October–February, 1 month ahead of the rainy season, is the ultimate forcing factor driving the changes of the CA rainy-season and annual δ^18^O_p_ variation. When the precession-induced October–February insolation strengthens (weakens), the tropospheric temperature over the CA rises (falls), and the water vapour transported to the CA increases (decreases) by the westerly circulation carrying water vapour of more (less) positive δ^18^O_v_ upstream, which leads to the enrichment (depletion) of CA δ^18^O_p_ under the combined action of the temperature effect and upstream circulation effect.

The SA annual δ^18^O_p_ variation primarily depends on the rainy-season (June–September) δ^18^O_p_. The main driving factor is attributed to the April–July insolation in the NH mid-low latitudes, 2 months ahead of the rainy season. When the April–July insolation is enhanced (reduced) by the precession forcing, the convection and precipitation in the SA monsoon region and its upstream water vapour source region in the tropical Indian Ocean are enhanced (depressed), along with the increase (decrease) in water vapour transport to the SA region from the upstream tropical Indian Ocean and Arabian Sea. As a consequence, the SA rainy-season and the resulting annual δ^18^O_p_ levels tend to be more negative (positive) due to the combined influence of precipitation amount effect and the upstream depletion effect.

Differently from the CA and SA regions, the change of the annual δ^18^O_p_ of the EA region is not determined by the whole monsoon-season δ^18^O_p_ (May–September) but that of the late-monsoon season (August–September), although there is also a certain contribution by the pre-monsoon season (April–May) to the annual δ^18^O_p_. At the orbital scale, the changes of the August–September and April–May δ^18^O_p_ are driven by the July–August insolation of the NH mid-low latitudes and the GIV, respectively. The enhanced July–August insolation can promote the August–September water vapour transport from the Arabian Sea and SA to EA, and lead to the reduced EA δ^18^O_p_ through the upstream depletion effect, while the retreat of the NH ice sheets helps increase the water vapour transport from the western Pacific to EA, resulting in the enriched EA δ^18^O_p_ in April–May. Therefore, the increase of the July–August insolation combined with the expansion of NH ice sheets is most conducive to depleting the EA δ^18^O_p_, while the decrease of insolation in combination with the retreat of the NH ice sheets is conducive to enriching the EA δ^18^O_p_.

The orbital-scale changes of the annual δ^18^O_p_ in the three regions of Asia all reflect the atmospheric and hydrologic responses to the precession forcing, although the specific physical processes involved are remarkably distinct. The current study confirmed some results from previous studies. For example, the CA δ^18^O_p_ variations indicated by the ice core δ^18^O records in the eastern Pamir [47] and the stalagmite δ^18^O record in Uzbekistan [11] are related to the temperature effect. However, the δ^18^O_p_ variation in SA is related to the precipitation amount effect. A comprehensive analysis based on stalagmite records in different regions of the world and climate model simulation results [53] indicates that there is a significant positive correlation between the δ^18^O_p_ values in the tropical monsoon regions, including India, and the local precipitation amount. Additionally, previous simulations of the δ^18^O_p_ during the last interglacial period [52], the last deglaciation period [32] and the Holocene [54] all show that the changes of δ^18^O_p_ in SA are mainly the result of the precipitation amount effect. Our results further indicate that the CA and SA δ^18^O_p_ variations are also affected by the upstream depletion effect associated with the changes in atmospheric circulation patterns, in addition to the local temperature or precipitation amount effect, and both are the results of the insolation changes caused by the precession forcing. The detailed physical processes of Asian orbital-scale δ^18^O_p_ changes (such as the vapour source location and transport pathway, repeated condensation, and re-evaporation) need to be further studied in the future.

A comprehensive understanding of the climatic significance of δ^18^O_p_ has been a challenge for the EA paleo-environment research for many years. Similar to some previous research results (e.g. [22,23]), our results suggest that the orbital-scale EA δ^18^O_p_ variation does not depend on local precipitation or temperature, but is the result of the atmospheric circulation change driven by the precession forcing. However, previous studies mostly emphasized the contribution of the whole monsoon rainy season to the annual δ^18^O_p_ variation, while we found that the upstream depletion process in August–September during the late-monsoon season is the key factor that controls the EA annual δ^18^O_p_ changes. Our simulation results make it clear that the precession-induced upstream depletion process in August–September dominates the EA annual δ^18^O_p_ changes at the orbital scale. At the same time, the EA annual δ^18^O_p_ variation is also related to the fluctuation of GIV at the precession scale. Although previous studies (e.g. [59]) speculated that the stalagmite δ^18^O records in EA could contain the information of winter temperature, our simulation finds that the expansion or contraction of the NH ice sheets (GIV) in the precession band mainly affects the EA April–May δ^18^O_p_ by modulating the Asia–Pacific large-scale atmospheric circulation.

This current study emphasizes that the climatic implications of the δ^18^O_p_ vary from place to place at the orbital scale. There are significant differences not only among the three regions of CA, SA and EA of Asia, but also between different sub-regions of the same climate type. For example, with the Pamir Plateau-Tianshan Mountains as the boundary, the Asian inland arid area can be roughly divided into two sub-regions—the western side is the arid region in CA, while the eastern side is the arid region in EA centred on the Tarim Basin. The CA and EA arid sub-regions are dominated respectively by the winter-rain and summer-rain regime climates [60]. Although the arid regions of CA and EA are all controlled by the westerly circulation, the climatic significance indicated by the annual δ^18^O_p_ variation may be different due to the difference in precipitation seasonality between these two regions. Additionally, the EA monsoon region selected in this study includes south-eastern China in its southern part. On the orbital timescale, precipitation in EA often has the characteristic of opposite variation patterns between the southern and northern EA [61]. The water vapour sources for the southern and northern EA are not exactly the same [62,63], and the proportion of water vapour directly from the ocean is higher in the southern part than that in the northern part [56,57]. Therefore, the interpretation of the orbital-scale δ^18^O_p_ variation for the southern part of EA monsoon region cannot be simply extended to the northern part, and additional analysis is needed to better understand the δ^18^O_p_ variation pattern for each specific sub-region.

The δ^18^O_p_ variations from our long-term transient simulations are helpful to deepen our understanding of the climatic implications of the oxygen isotope records of the Asian stalagmite and other sedimentary deposits by direct comparisons between the simulation results and geological records. However, due to the lack of available geological records, only the simulated EA annual δ^18^O_p_ series and a Chinese stalagmite δ^18^O record [8] in the past 300 kyr can be well compared in detail (Note S3). At present, limited by the time span and continuity, the stalagmite δ^18^O records in CA [11] and SA [10] are not sufficient for direct comparisons with our simulation results. Therefore, the phase differences between the CA, SA and EA δ^18^O_p_ series obtained by our simulation are currently difficult to verify with geological records. We also noted that the phase difference between the orbital-scale evolution series of the EA summer monsoon and the Indian summer monsoon reconstructed with the multi-proxy records from the Arabian Sea [64] are significantly different due to the phase difference between our simulated EA and SA annual δ^18^O_p_ series. These discrepancies deserve further investigation in the future.

## Supplementary Material

nwac182_Supplemental_File
