# Supplementary material for: Model-based orbital-scale precipitation δ18O variations and distinct mechanisms in Asian monsoon and arid regions
Source: Natl Sci Rev. 2022 Aug 30;9(11):nwac182. doi: 10.1093/nsr/nwac182 (PMC9647003; doi:10.1093/nsr/nwac182)
Supplement: nwac182_Supplemental_File [file nwac182_supplemental_file.pdf]

Supplementary Data for

Model-based orbital-scale precipitation δ^{18}O variations and distinct mechanisms in Asian monsoon and arid regions

Xiaodong Liu[1,3*], Xiaoxun Xie[1], Zhengtang Guo[2,3], Zhi-Yong Yin[4] and Guangshan Chen[1]

[1] SKLLQG, Institute of Earth Environment, Chinese Academy of Sciences, Xi'an 710061, China

[2] Institute of Geology and Geophysics, Chinese Academy of Sciences, Beijing 100029, China

[3] University of Chinese Academy of Sciences, Beijing 100049, China

[4] Department of Environmental & Ocean Sciences, University of San Diego, San Diego, CA 92110, USA

[*] Corresponding author (email: liuxd@loess.llqg.ac.cn)

Contents

Note S1. Numerical model and analysis methods ..…………………………………...3

Note S2. Validations of the annual cycles of the simulated air temperature, precipitation and $\delta^{18}O_p$ ……….………...…………………………………...5

Note S3. Comparison of the simulated $\delta^{18}O_p$ and Chinese stalagmite records …..…...6

Note S4. Determination of the CA, SA and EA rainy seasons ………………...…...7

Note S5. Comparisons of periodicities of the GIV, GHG, and annual and rainy-season insolation series …………………………..…………………………………...8

Note S6. PCA of the CA January-December tropospheric temperature series ……......9

Note S7. Correlations between the CA rainy-season $\delta^{18}O_p$, tropospheric temperature and $\delta^{18}O_v$ ……………….………………..…………………………………...10

Note S8. Correlations of the CA 700-hPa temperature and the tropospheric $\delta^{18}O_v$ with the 45°N insolation …………………..…………………………………...11

Note S9. PCA of the SA January-December $\delta^{18}O_p$ series …………………………...12

Note S10. Correlation patterns of the $\delta^{18}O_v$ field with SA $\delta^{18}O_p$ series during glacial and interglacial periods ..……………………..……………………………...13

Note S11. Correlations of the SA precipitation, $\delta^{18}O_p$ and the source region $\delta^{18}O_v$ with the 30 °N insolation ……………………..……………………………...14

Note S12. Regression analysis of the water vapor transport flux and content onto the 30 °N insolation ..…………………………..……………………………...15

Note S13. PCA and correlation analysis of the January-December EA $\delta^{18}O_p$ series ..………………………………..……………………………...16

Note S14. Correlations of August-September and April-May EA $\delta^{18}O_p$ with the EA $\delta^{18}O_v$ ..………………………………..……………………………...18

Note S15. Correlations of the insolation, GIV and GHGs with the August-September and April-May EA $\delta^{18}O_p$ and water vapor content ..…………………………..19

References ………………………………………….....………………….…...20

Note S1. Numerical model and analysis methods

The National Center for Atmospheric Research Community Climate System Model version 3 (CCSM3) used in this study is a three-dimensional climate system model with dynamically coupled atmosphere, ocean, land and sea ice components. The atmosphere module is the third edition of the Community Atmosphere Model (CAM3), with ~3.75° horizontal resolution (T31) and 26 hybrid coordinate levels in the vertical direction. In this study, a continuous integration was performed from 300 ka (kilo annum before present, the same below) to 0 ka by using 100-time acceleration of orbital forcing (similar to Kutzbach et al. [1]; Xie et al. [2]). In this transient simulation, various external forcing conditions, such as the Earth's orbital forcing, greenhouse gases (GHGs) and global ice sheets, kept varying with time. The actual Earth's orbital parameters in the geological period [3] were employed to calculate the astronomical solar radiation at each time step, and the atmospheric CO_2 and CH_4 concentrations were assigned according to the GHG series reconstructed from the Antarctic ice cores [4]. The Earth's orbital parameters and the GHGs were advanced by 100 years at the end of each model year. In the integration process, the Earth's ice sheet status at each time step was set according to the continuous records of the marine $\delta^{18}O$ reflecting the global ice volume (GIV) [5] and the reconstructed ice sheet distributions of millennium resolution since the past 21 kyr (ICE-5G, [6]). The continental ice sheets were reconfigured at steps of an equivalent 40-m sea level rise or fall, and the changes in ice sheets were accompanied by the corresponding sea-level changes and reconfigurations of land-ocean distribution. In fact, the change of the GIV since the mid-Pleistocene was mainly caused by the fluctuation of the NH ice sheets [7]. Fig. S1 shows the time series of the major climate forcing factors driving CAM3 for the last 300-kyr transient simulation, including Earth's orbit parameters, GIV, atmospheric greenhouse gas concentrations (as equivalent CO_2 concentration), and global tropical mean sea surface temperature.

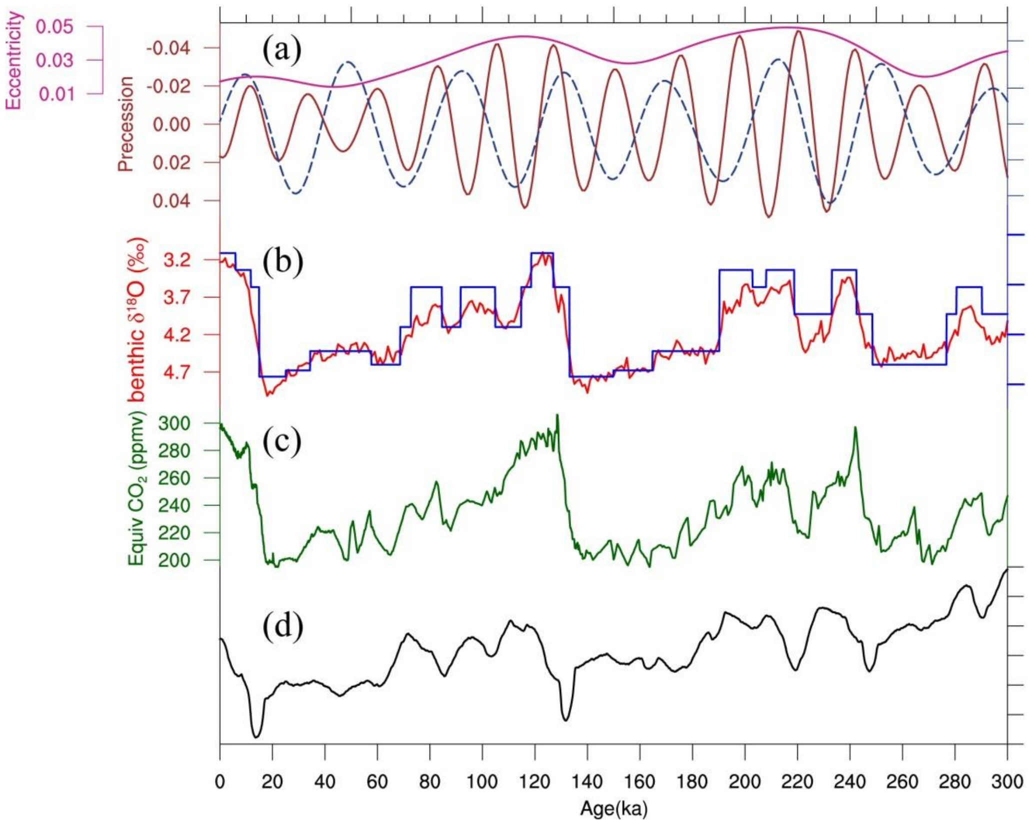

Fig. S1 Time series of main climate forcing factors for the last 300-kyr transient simulation. (a) Orbital parameters [3]: precession (brown), obliquity (royalblue) and eccentricity (pink); (b) The LR04 stacked benthic $\delta^{18}O$ (red, [5]) and the phased GIV change (blue) used in this transient simulation; (c) Equivalent CO_2 level [4]; (d) Global tropical mean sea surface temperature.

A series of multivariate statistical methods were used for analysis of the simulation results. The periodicity of climate variables was identified by employing power spectrum analysis [8]. Cross spectrum analysis [9] was used to reveal the phase relationships among the $\delta^{18}O_p$ of different regions and forcing factors, such as the insolation variation of different months due to the orbital parameters. Principal component analysis (PCA) [10] was used to identify major modes of simultaneous variation patterns among multiple variables that have various degrees of correlations among them. We used the Pearson's correlation analysis to explore the associations and potential causal relationships between different variables [11], while partial correlation was used to evaluate the strength of the relationship between two variables as the effects of other variables (control variables) are eliminated [12]. In order to reveal the spatial response pattern of a given variable to possible influencing factors,

we used regression analysis [13] to identify the empirical relationships first and then mapped the regression coefficients representing the changes in the dependent variable corresponding to per-unit change of the independent variable.

Note S2. Validations of the annual cycles of the simulated air temperature, precipitation and $\delta^{18}O_p$

Comparing with the modern observations of temperature (CRU temperature data) [14] and precipitation (CPC merged analysis of precipitation) [15], the annual cycles of the simulated 300kyr-averaged temperature and precipitation in the CA, SA and EA regions are generally consistent with their respective observational characteristics of the 30-year (1981-2010) averages (Fig. S2), although the simulated monthly temperatures are consistently lower than the observations due to higher atmospheric CO_2 content at the present day and the presence of the Quaternary glacial periods. The simulated mean annual cycles of the $\delta^{18}O_p$ values in the three study regions are also similar to the limited modern observations in Central Asia (e.g., [16]), South Asia (e.g., [17]), and East Asia (e.g., [18]), although the simulated annual variation amplitudes are relatively small. These long-term mean annual cycles of temperature, precipitation and $\delta^{18}O_p$ in the three regions are relatively stable, even in different glacial and interglacial stages (figures omitted).

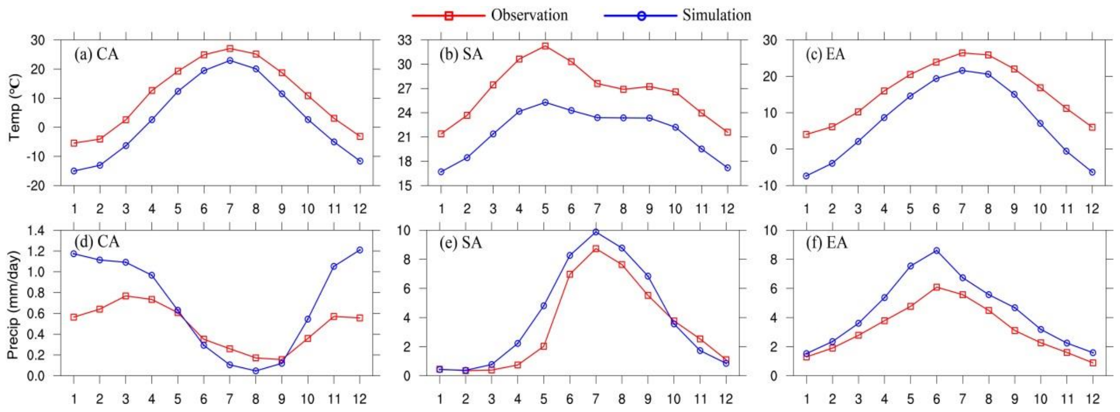

Fig. S2 Annual cycles of air temperature (a, b, c) and precipitation (d, e, f) averaged for the simulated past 300 kyr (blue lines) and the observed during the past 30 years (red lines) in the CA (a, d), SA (b, e) and EA (c, f) regions.

Note S3. Comparison of the simulated $\delta^{18}O_p$ and Chinese stalagmite records

The stalagmite $\delta^{18}O$ ($\delta^{18}O_c$) is jointly controlled by $\delta^{18}O$ of dripwater and the temperature inside the cave [19]. Since the dripwater $\delta^{18}O$ mainly depend on the local precipitation of $\delta^{18}O$ ($\delta^{18}O_p$) and the temperature- dependent fractionation is relatively small, $\delta^{18}O_c$ can inherit the rain water $\delta^{18}O_p$ to a large extent [20,21]. Here, we attempted to compare the simulated precipitation $\delta^{18}O_p$ series with relevant geological records. We only considered those stalagmite $\delta^{18}O$ records with long time spans, high temporal resolutions, and reliable dating from China in the EA region [22]. Since the variation amplitudes of the simulated $\delta^{18}O_p$ series at the orbital scale are relatively small, similar to some of the previous studies [23–26], the simulated EA annual $\delta^{18}O_p$ series and the Chinese stalagmite oxygen isotope records are first standardized and then compared (Fig. S3). The result indicates that the variations of the oxygen isotope

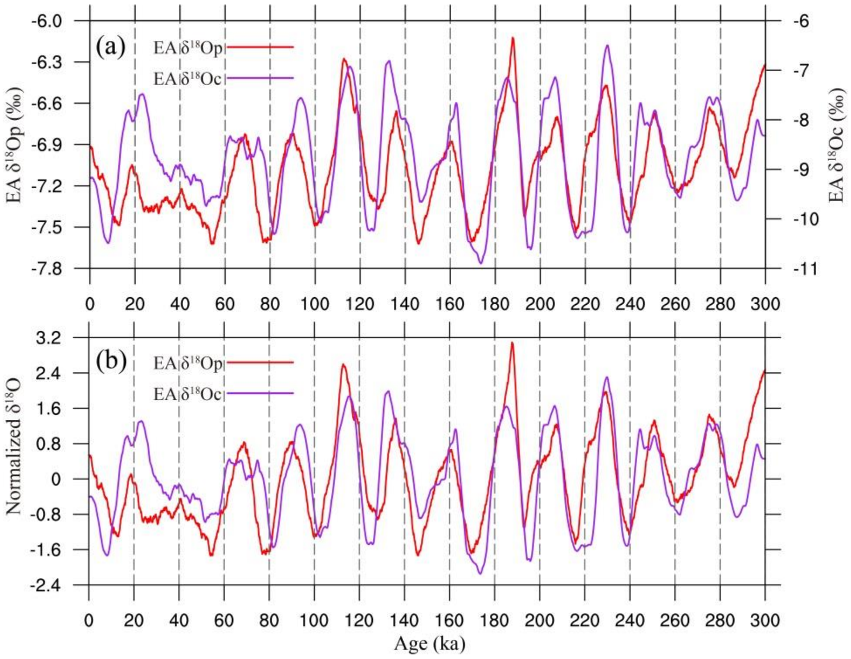

Fig. S3 (a) Comparison between the EA annual $\delta^{18}O_p$ series from the simulation and the Chinese stalagmite $\delta^{18}O_c$ series [22] over the past 300 kyr; (b) Same as (a) but for the standardized series.

composition in the simulated and geological records are highly consistent during the past 300 kyr (r=0.647), and the corresponding cross-spectral analysis also indicates that both of them vary almost in phase at the 23-kyr precession band, with only a 0.6-kyr phase difference. This also partially verifies the reliability of our simulation results, suggesting that the model simulated $\delta^{18}O_p$ values are highly comparable to the geological records after considering all major atmospheric and hydrological processes that affect the fractionation of oxygen isotope, at least for the EA region.

Note S4. Determination of the CA, SA and EA rainy seasons

Considering that the annual $\delta^{18}O_p$ is obtained as the average of individual month's $\delta^{18}O_p$ in the whole year weighted by the precipitation amount of each month, the annual $\delta^{18}O_p$ usually depends on the rainy-season $\delta^{18}O_p$. Therefore, it is necessary to specify the rainy season for each study region for analyzing the changes in the annual $\delta^{18}O_p$. By calculating the percentages of the monthly precipitation and the product (RD) of mean monthly precipitation and monthly $\delta^{18}O_p$ from January to December in the past 300 kyr (Table S1), it is found that high RD values occur during November-March for the CA region, June-September for the SA region, and May-September for the EA region, accounting for 81.7%, 73.6%, and 72.8% of their annual totals, respectively, and hence the respective rainy seasons are defined.

Table S1 Percentages (%) of monthly and rainy-season (corresponding to the months shaded in green in the rows of each region) precipitations (R) and products of precipitation and $\delta^{18}O_p$ (RD) accounting for their contributions to the annual totals (the Rainy Season column), averaged for the past 300 kyr in the Central Asia (CA), South Asia (SA) and East Asia (EA) regions.

		Jan	Feb	Mar	Apr	May	Jun	Jul	Aug	Sep	Oct	Nov	Dec	Rainy Season
CA	R	14.4	12.3	13.4	11.5	7.7	3.5	1.3	0.6	1.4	6.7	12.5	14.8	**67.4**
	RD	20.3	15.2	11.8	6.4	3.2	1.5	0.7	0.3	0.8	5.3	13.9	20.5	**81.7**
SA	R	0.9	0.7	1.6	4.5	10	16.7	20.6	18.3	13.8	7.4	3.5	1.8	**69.4**
	RD	0.3	0.3	0.8	3.3	8.5	14.7	19.1	20.9	18.9	9.3	2.8	1	**73.6**
EA	R	2.9	4.1	6.9	10	14.5	16	12.9	10.7	8.7	6.1	4.2	3	**62.8**
	RD	2.7	3	3.5	4.3	10.5	18.2	18.5	15.2	10.4	6.2	4.3	3.3	**72.8**

Note S5. Comparisons of periodicities of the GIV, GHG, and annual and rainy-season insolation series

Power spectral analysis results of the climate forcing factors in the late Quaternary (Fig. S4) indicate that the variations of GIV [5] and GHGs (as the CO_2 equivalent concentration; [4]) are dominated by the quasi-100-kyr cycles (Fig. S4a,b), which also include the signals of the 41-kyr and 23-kyr cycles related respectively to the Earth's orbital obliquity and precession [27]. In the meantime, while the mid- to low-latitude rainy-season insolation has significant 23-kyr cycles (Fig. S4d-f), the annual average insolation has the dominant 41-kyr cycle rather than the 23-kyr cycle (Fig. S4c).

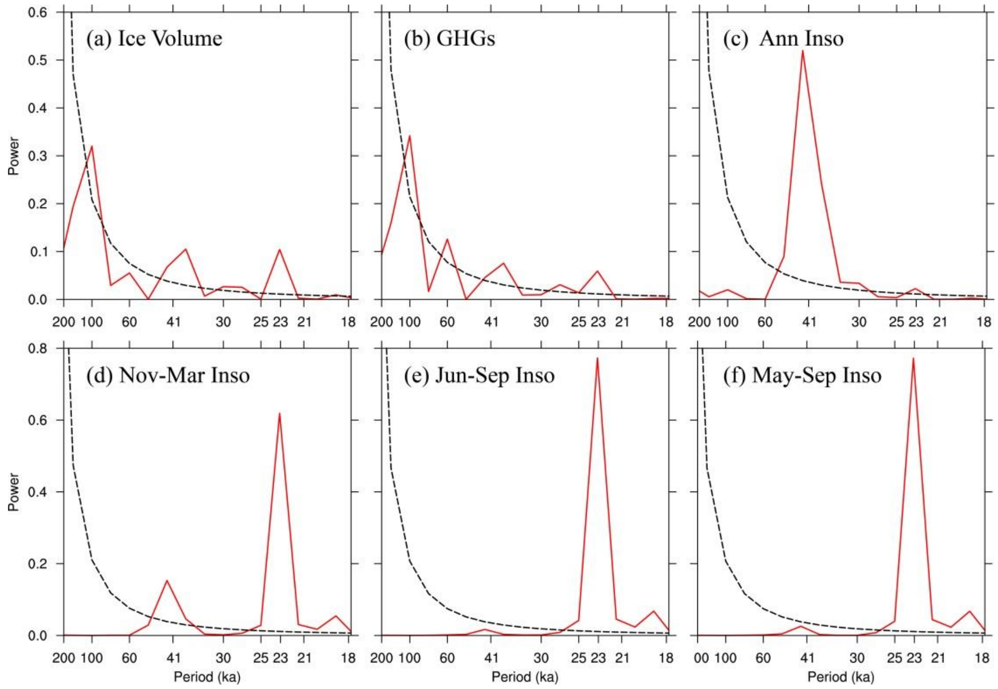

Fig. S4 Power spectrum analysis results of the global ice volume ([5]; a), reconstructed concentration of greenhouse gases ([4], b), and annual (c), November-March (d), June-September (e) and May-September (f) mean 30 °N insolation series over the past 300 kyr. The black dashed lines indicate the red noise spectrum with 90% confidence level.

Note S6. PCA of the CA January-December tropospheric temperature series

In order to establish the relationships among the individual monthly $\delta^{18}O_p$ and temperature series, we first extracted the most dominant temporal variation patterns of the CA January-December temperature series at various isobaric levels from the surface to the upper troposphere as represented by the first principal component (PC1) using PCA. The variance contributions of PC1 for different levels are mostly between 45-50% (Table S2). From the surface to 300 hPa, PC1 of each level mainly reflects a consistent variation pattern of the temperature from October to February, closely matching the November-March rainy season, and a reversed variation pattern of the temperature in the warm-season months (Table S2). In other words, the most dominant temperature variation pattern shows that when the PC1 scores are positive, there is the tendency for the surface to 300-hPa temperatures to be above normal during the cold-season months and below normal during the warm-season months. When the PC1 scores are negative, the opposite temperature variation pattern occurs.

Table S2 The PC loadings of the first principal component (PC1) and its variance contribution (VC, %) from PCA of the January- December temperature variation series at isobaric levels from the surface to 200 hPa in the CA region during the past 300 kyr. The loadings of PC1 greater than 0.5 are in boldface.

	PC1												VC
	Jan	Feb	Mar	Apr	May	Jun	Jul	Aug	Sep	Oct	Nov	Dec	
200hPa	**0.686**	**0.889**	**0.968**	**0.946**	**0.643**	0.269	-0.118	-0.652	-0.797	-0.567	-0.037	0.400	42.8
300hPa	**0.883**	**0.545**	-0.060	-0.536	-0.809	-0.926	-0.865	-0.510	0.203	**0.738**	**0.828**	**0.905**	49.8
400hPa	**0.918**	**0.764**	0.154	-0.313	-0.624	-0.83	-0.839	-0.572	0.089	**0.718**	**0.825**	**0.888**	47.1
500hPa	**0.922**	**0.796**	0.229	-0.196	-0.513	-0.753	-0.775	-0.506	0.145	**0.778**	**0.859**	**0.909**	45.4
600hPa	**0.931**	**0.827**	0.341	-0.038	-0.381	-0.674	-0.728	-0.441	0.217	**0.838**	**0.908**	**0.939**	45.4
700hPa	**0.941**	**0.878**	**0.522**	0.181	-0.194	-0.549	-0.651	-0.390	0.269	**0.873**	**0.938**	**0.957**	45.9
850hPa	**0.955**	**0.945**	**0.812**	0.512	0.099	-0.283	-0.411	-0.139	0.451	**0.876**	**0.957**	**0.967**	48.6
Surface	**0.954**	**0.950**	**0.831**	0.485	-0.001	-0.374	-0.476	-0.151	0.489	**0.892**	**0.975**	**0.961**	50.3

Note S7. Correlations between the CA rainy-season $\delta^{18}O_p$, tropospheric temperature and $\delta^{18}O_v$

We also calculated the correlation coefficients between the CA rainy-season $\delta^{18}O_p$ and the $\delta^{18}O_v$ of each isobaric level and the entire troposphere (300hPa-surface) (Table S3). High positive correlations are found between these variables. For example, the correlation coefficient between the $\delta^{18}O_p$ and the whole-troposphere $\delta^{18}O_v$ is 0.823, leading to the conclusion that the $\delta^{18}O_v$ is also an important influencing factor of the $\delta^{18}O_p$ since the inheritance in the water cycle. However, the correlations between the temperature of each isobaric level and the $\delta^{18}O_v$ at the same level are not as high. For example, the correlation coefficient between the 700-hPa temperature and $\delta^{18}O_v$ is only 0.090 (Table S3), suggesting that the tropospheric temperature and $\delta^{18}O_v$ can independently affect the $\delta^{18}O_p$ levels with little interactions in-between.

Table S3 Correlation coefficients between the CA rainy-season (November-March) precipitation $\delta^{18}O_p$, temperatures (T), and water vapor $\delta^{18}O_v$ at different isobaric levels, as well as for the whole troposphere (300hPa-surface).

	200hPa	300hPa	400hPa	500hPa	600hPa	700hPa	850hPa	925hPa	300hPa–Surface
$\delta^{18}O_p$~T	0.442	0.732	0.646	0.671	0.733	0.770	0.721	0.653	0.728
$\delta^{18}O_p$~$\delta^{18}O_v$	0.292	0.774	0.656	0.582	0.580	0.633	0.806	0.849	0.823
T~$\delta^{18}O_v$	0.781	0.496	0.384	0.133	0.030	0.090	0.262	0.309	0.326

Note S8. Correlations of the CA 700-hPa temperature and the tropospheric $\delta^{18}O_v$ with the 45 °N insolation

Table S4 presents the correlations between the January-December monthly temperatures at 700 hPa in the CA region and the insolation at approximately the same latitudes (45 °N). It can be seen that the correlation coefficients between the monthly average temperature and the leading 1-2 month insolation are usually the highest. For example, the correlation coefficient between the 700-hPa December temperature and the leading November insolation is 0.569. Therefore, we examined the correlation between the CA rainy-season (November-March) average temperature and the mean insolation leading by 1 month (October-February). The result shows that the correlation coefficient between the CA average rainy-season 700-hPa temperature and the average October-February insolation is 0.619 (Table S5), which is significantly higher than those in the lower troposphere below 700 hPa, while the strengths of the correlations maintain for the levels up to 300 hPa (Table S5). Since the focus of this analysis is the potential linkage between the insolation and temperature for the months and isobaric levels relevant to the CA rainy season, those stronger correlations in the summer months (Table S4) or those in the upper troposphere isobaric levels with less moisture content (Table S5) are excluded. Similarly, the correlation coefficients (Table S5) between the October-February average insolation and the CA rainy-season $\delta^{18}O_v$ in different isobaric levels of the troposphere indicate that the $\delta^{18}O_v$ is strongly associated with the insolation variations. For example, the correlation between the whole-troposphere $\delta^{18}O_v$ and insolation is as high as 0.877 (Table S5). It is noted that the correlation of S_{10_2} with the rainy-season surface $\delta^{18}O_{v11\_3}$ is strong with r=0.883. In contrast, there is no correlation between the rainy-season precipitation and insolation (r=−0.048), which once again shows that the CA precipitation isotopic composition is related to the water vapor transport rather than the local precipitation variation.

Table S4 Correlation coefficients of the January-December monthly (T_1, T_2…T_{12}) and November-March average (T_{11_3}) 700-hPa temperature series in the CA region with the January-December monthly (S_1, S_2…S_{12}) and October-February average (S_{10_2}) 45 °N insolation series. The boldface values indicate the correlation coefficients between the temperature and the one-month-leading insolation series.

	T_1	T_2	T_3	T_4	T_5	T_6	T_7	T_8	T_9	T_{10}	T_{11}	T_{12}	T_{11_3}
S_1	0.540	**0.526**	0.309	0.086	-0.217	-0.574	-0.668	-0.588	-0.175	0.398	0.495	0.491	0.525
S_2	0.351	0.466	**0.592**	0.580	0.345	-0.180	-0.495	-0.783	-0.651	-0.058	0.166	0.205	0.371
S_3	-0.167	0.026	0.508	**0.778**	0.830	0.525	0.187	-0.373	-0.780	-0.615	-0.381	-0.330	-0.113
S_4	-0.450	-0.271	0.273	0.630	**0.861**	0.829	0.607	0.057	-0.599	-0.772	-0.603	-0.569	-0.392
S_5	-0.613	-0.485	-0.007	0.353	0.698	**0.930**	0.870	0.448	-0.304	-0.751	-0.675	-0.667	-0.564
S_6	-0.636	-0.606	-0.343	-0.080	0.299	0.779	**0.936**	0.791	0.142	-0.510	-0.573	-0.597	-0.613
S_7	-0.330	-0.449	-0.616	-0.625	-0.402	0.152	0.516	**0.831**	0.683	0.093	-0.132	-0.183	-0.354
S_8	0.261	0.072	-0.434	-0.733	-0.851	-0.656	-0.360	0.215	**0.745**	0.683	0.452	0.415	0.206
S_9	0.567	0.428	-0.055	-0.409	-0.721	-0.907	-0.834	-0.382	0.378	**0.753**	0.648	0.641	0.519
S_{10}	0.642	0.574	0.232	-0.068	-0.441	-0.850	-0.951	-0.708	0.011	0.608	**0.624**	0.638	0.611
S_{11}	0.629	0.617	0.406	0.175	-0.200	-0.708	-0.905	-0.836	-0.247	0.443	0.547	**0.569**	0.612
S_{12}	**0.589**	0.589	0.406	0.193	-0.150	-0.607	-0.774	-0.743	-0.277	0.387	0.512	0.520	0.578
S_{10_2}	0.638	0.619	0.380	0.132	-0.244	-0.731	-0.902	-0.804	-0.207	0.474	0.567	0.585	**0.619**

Table S5 Correlation coefficients of the October-February average 45 °N insolation (S_{10_2}) with the November-March average temperatures (T_{11_3}, the first row) and the corresponding water vapor $\delta^{18}O_{v11\_3}$ (the second row) at the surface and different isobaric levels, as well as for the whole-troposphere (300hPa-surface) average during the CA rainy season.

	200hPa	300hPa	400hPa	500hPa	600hPa	700hPa	850hPa	925hPa	Surface	300hPa -Surface
$S_{10\_2} \sim T_{11\_3}$	0.420	0.795	0.660	0.647	0.648	0.619	0.498	0429	0.500	0.603
$S_{10\_2} \sim \delta^{18}O_{v11\_3}$	0.250	0.809	0.807	0.729	0.692	0.741	0.889	0.932	0.883*	0.877

Note S9. PCA of the SA January-December $\delta^{18}O_p$ series

To reveal the dominant temporal variation pattern in the SA $\delta^{18}O_p$ data, we performed PCA on the January-December $\delta^{18}O_p$ series to characterize how $\delta^{18}O_p$ varies in different months in the SA region during the past 300 kyr. The results show that PC1, accounting for approximately 50% of the total variance, mainly reflects the consistent variation pattern of $\delta^{18}O_p$ during June-September (Table S6), matching the SA rainy or monsoon season. In the meantime, PC1 also represents the $\delta^{18}O_p$ anomalies of opposite signs for the winter-spring season (January-April) (Table S6). In other words, when the PC1 scores are positive, the SA rainy-season $\delta^{18}O_p$ values

tend to be above normal, while the winter-spring season will have below-normal $\delta^{18}O_p$ values. When the PC1 scores are negative, the opposite pattern happens.

Table S6 The PC loadings of the first and second principal components (PC1 and PC2) and their variance contributions (VC, %) from PCA on the January-December $\delta^{18}O_p$ variation series in the SA region during the past 300 kyr. The boldface values indicate the four largest positive loadings of PC1.

	Jan	Feb	Mar	Apr	May	Jun	Jul	Aug	Sep	Oct	Nov	Dec	VC
PC1	-0.583	-0.828	-0.856	-0.890	-0.328	**0.797**	**0.909**	**0.930**	**0.914**	0.099	-0.312	-0.106	49.5
PC2	0.789	0.568	-0.213	-0.596	-0.828	-0.949	-0.882	-0.621	0.097	0.609	0.712	0.667	16.6

Note S10. Correlation patterns of the $\delta^{18}O_v$ field with SA $\delta^{18}O_p$ series during glacial and interglacial periods

In order to examine the robustness of the relationship of the SA $\delta^{18}O_p$ to the water vapor isotope field, the past 300 kyr were divided into five periods according to the marine oxygen isotope stages (MIS; [5]), including three glacial periods (MIS2-4, MIS6, MIS8) and two interglacial periods (MIS5, MIS7) (Fig. S5). It is found that the spatial patterns of the correlations between the whole-troposphere $\delta^{18}O_v$ field and SA $\delta^{18}O_p$ series are very similar in both glacial and interglacial periods of the past 300 kyr (Fig. S6), which also indirectly verifies the validity of the upstream water vapor source region specified for the SA rainy-season precipitation.

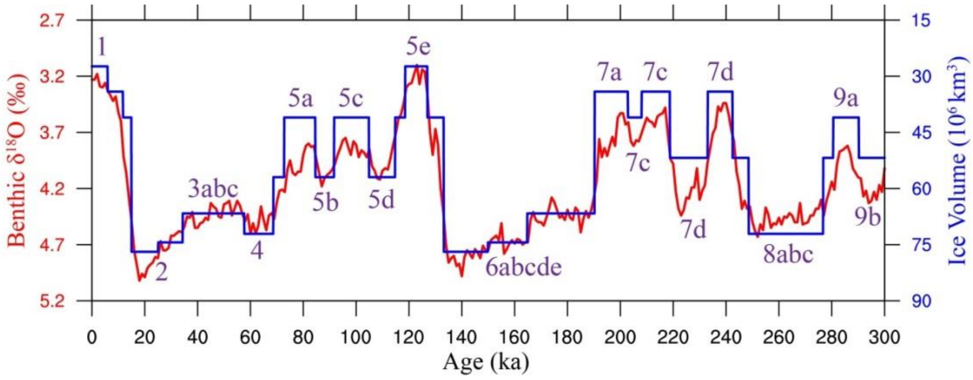

Fig. S5 The GIV variation (red line) represented by the marine benthic foraminifera δ^{18}O stacks in the past 300 kyr [5] and the phased GIV change (blue line) used in this transient simulation. The numbers and letters in the figure represent the marine isotope stages (MIS).

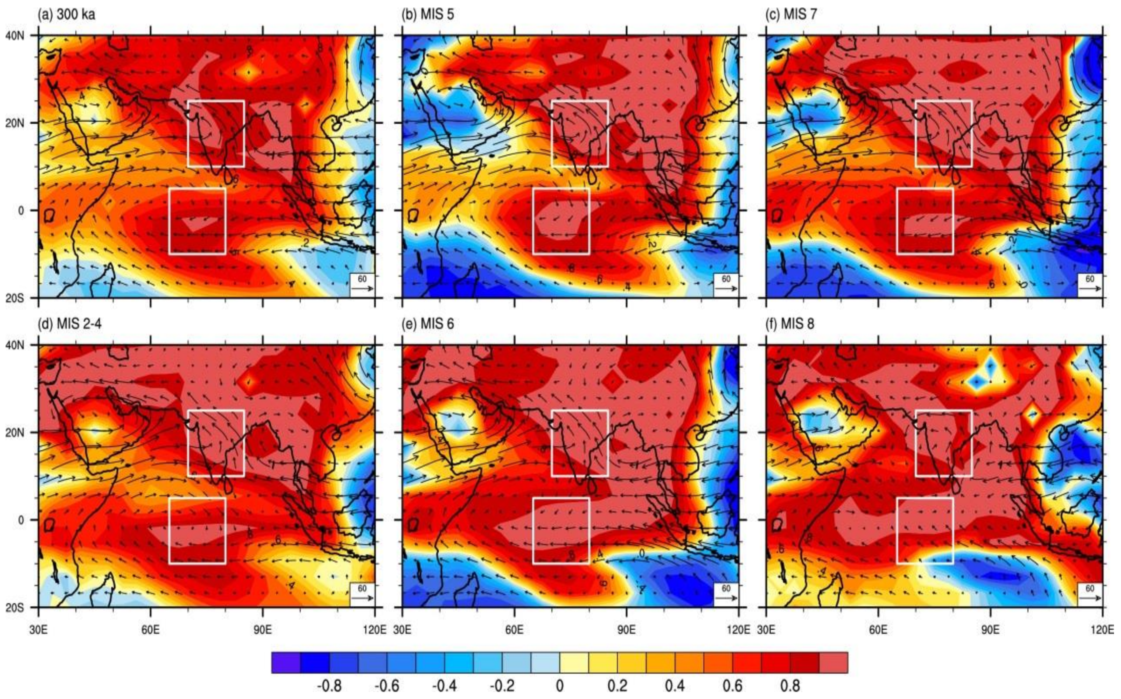

Fig. S6 The correlation field (shaded) of the whole-troposphere (300hPa-surface) water vapor δ^{18}O$_v$ with the SA rainy-season precipitation δ^{18}O$_p$ series and the regression coefficient field (arrows, unit: kg/m/s) of the whole-troposphere water vapor flux in the Asia-Indian Ocean region with the SA rainy-season −δ^{18}O$_v$ series for the past 300 kyr as the independent variable (a), MIS5 (b), MIS7 (c), MIS2-4 (d), MIS6 (e) and MIS8 (f). The white boxes are the same as in Fig. 5 in the main text.

Note S11. Correlations of the SA precipitation, δ^{18}O$_p$ and the source region δ^{18}O$_v$ with the 30 °N insolation

To confirm the linkage between the variation of the SA rainy-season precipitation and the precession cycle, we correlated the January-December monthly precipitation series with the monthly insolation at 30 °N. Table S7 shows that the precipitation of each month in the rainy season is usually most closely related to the insolation leading by two months. For example, the highest correlation coefficient of

June (September) precipitation is with April (July) 30 °N insolation as 0.827 (0.736). Thus, the positive correlations between SA June-September precipitation and April-July insolation tend to be highest, while the negative correlations between the $\delta^{18}O_p$ or source region $\delta^{18}O_{vs}$ during the SA rainy season and April-July insolation are also the strongest (Table S7).

Table S7 Correlation coefficients of the January-December monthly 30 °N insolation series (S_1, S_2…S_{12}) with the SA January-December monthly precipitation (R_1, R_2…R_{12}), rainy-season precipitation (R_{6_9}), precipitation $\delta^{18}O_p$, and the whole- troposphere water vapor oxygen isotope ratio in the upstream source region ($\delta^{18}O_{vs}$). The boldface values show that the precipitation, $\delta^{18}O_p$ or $\delta^{18}O_{vs}$ series during June- September has the highest correlations with the corresponding two-month-leading insolation series.

	S_1	S_2	S_3	S_4	S_5	S_6	S_7	S_8	S_9	S_{10}	S_{11}	S_{12}
R_1	0.123	-0.122	-0.298	-0.335	-0.285	-0.151	0.066	0.270	0.309	0.250	0.193	0.181
R_2	0.593	0.285	-0.122	-0.381	-0.526	-0.541	-0.353	0.049	0.382	0.528	0.583	0.621
R_3	0.686	0.723	0.470	0.153	-0.180	-0.503	-0.691	-0.521	-0.098	0.258	0.482	0.593
R_4	0.474	0.762	0.753	0.552	0.235	-0.187	-0.623	-0.775	-0.505	-0.138	0.148	0.306
R_5	0.178	0.683	0.920	0.850	0.579	0.111	-0.491	-0.897	-0.791	-0.468	-0.181	-0.024
R_6	-0.250	0.275	0.697	**0.827**	0.749	0.452	-0.078	-0.634	-0.803	-0.689	-0.523	-0.417
R_7	-0.379	0.051	0.461	0.641	**0.656**	0.488	0.100	-0.394	-0.632	-0.626	-0.547	-0.493
R_8	-0.473	-0.216	0.147	0.395	0.540	**0.550**	0.341	-0.092	-0.432	-0.555	-0.560	-0.529
R_9	-0.553	-0.757	-0.631	-0.345	0.017	0.421	**0.736**	0.674	0.256	-0.137	-0.372	-0.449
R_{10}	0.027	-0.482	-0.806	-0.842	-0.681	-0.304	0.278	0.789	0.826	0.593	0.350	0.213
R_{11}	0.229	-0.163	-0.480	-0.588	-0.554	-0.367	0.004	0.430	0.593	0.534	0.422	0.351
R_{12}	0.164	0.032	-0.091	-0.145	-0.160	-0.132	-0.049	0.071	0.140	0.152	0.157	0.178
R_{6_9}	-0.471	-0.105	0.317	**0.557**	**0.651**	**0.575**	**0.259**	-0.246	-0.574	-0.649	-0.614	-0.565
$\delta^{18}O_p$	0.570	0.168	-0.328	**-0.633**	**-0.775**	**-0.718**	**-0.361**	0.254	0.674	0.780	0.737	0.670
$\delta^{18}O_{vs}$	0.735	0.481	0.002	**-0.380**	**-0.655**	**-0.778**	**-0.613**	-0.078	0.442	0.699	0.776	0.768

Note S12. Regression analysis of the water vapor transport flux and content onto the 30 °N insolation

To explain the insolation-induced atmospheric physical processes affecting the SA precipitation and the source-region $\delta^{18}O_v$, Fig. S7 shows the regression coefficient fields of the June-September whole-troposphere water vapor transport flux (Fig. S7a)

and atmospheric water vapor content (Fig. S7b) in the Asia-Indian Ocean region with the April-July average 30°N insolation as the independent variable. When the insolation is strengthened in the NH spring and summer, the water vapor transport into the SA from the southwest is enhanced (Fig. S7a), resulting in increases in water vapor content (Fig. S7b) and precipitation over the SA region.

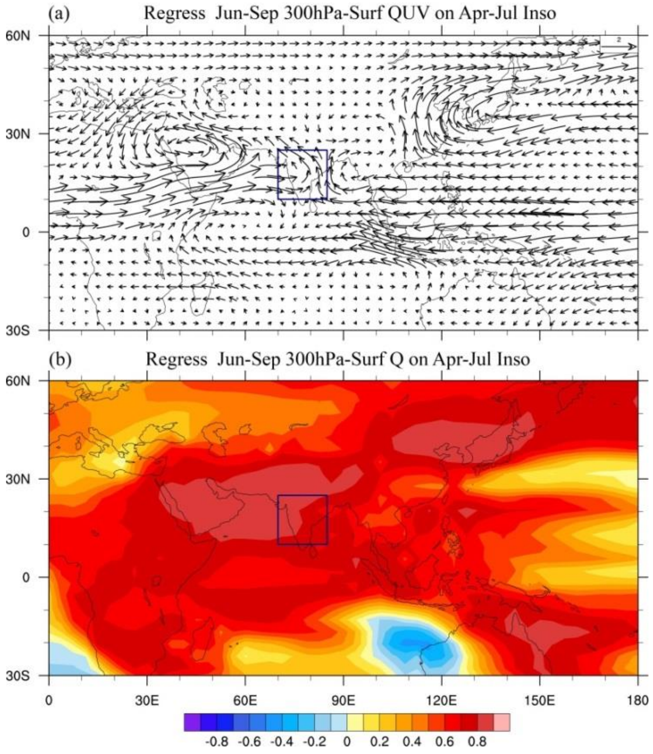

Fig. S7 Regression coefficient fields of the whole-troposphere (300hPa-surface) water vapor flux (a, unit: kg/m/s) and atmospheric water vapor content (b, unit: kg/m^2) in the Asia-Indian Ocean region with the April-July average 30°N insolation as the independent variable. The dark blue boxes indicate the SA region.

Note S13. PCA and correlation analysis of the January-December EA $\delta^{18}O_p$ series

Using PCA on January-December EA $\delta^{18}O_p$ series (Table S8), we identified an inversed variation pattern between the months of late summer to early autumn (August-September) and those of late spring (April-May), represented by PC1 that accounts for approximately 54% of the total variance. Although the PC loadings of the October-March $\delta^{18}O_p$ series are also relatively strong, these non-rainy-season

months account for less than 27.2% of the annual total (Table S1) and, therefore, their contributions to the annual $\delta^{18}O_p$ variation should be limited. The second principal component (PC2) only accounts for 18.2% of the total variance, and the months with high loading values are limited to May-June (Table S8). The correlations among the EA $\delta^{18}O_p$ series in different months from January to December indicate that the variation patterns between August and September or those between April and May are highly consistent, with the corresponding correlation coefficients of 0.855 and 0.690, respectively (Table S9). Thus, August-September and April-May $\delta^{18}O_p$, as indicated by their loadings of PC1, may contribute most to the variation in the EA annual $\delta^{18}O_p$.

Table S8 Same as Table S6 but for the $\delta^{18}O_p$ variation series in the EA region.

	Jan	Feb	Mar	Apr	May	Jun	Jul	Aug	Sep	Oct	Nov	Dec	VC
PC1	0.895	0.895	0.631	-0.394	-0.736	-0.351	0.482	0.739	0.801	0.853	0.889	0.855	54.1
PC2	-0.146	0.259	0.598	0.322	-0.268	-0.849	-0.826	-0.173	0.019	0.101	-0.194	-0.294	18.2

Table S9 Correlation coefficients between the EA January-December monthly precipitation $\delta^{18}O_p$ series ($\delta^{18}O_{p1}$, $\delta^{18}O_{p2}$ $\cdots$$\delta^{18}O_{p12}$) for the past 300 kyr. The boldface values indicate the correlation coefficients between the May-September $\delta^{18}O_p$ with the corresponding one-month-leading $\delta^{18}O_p$ series.

	$\delta^{18}O_{p1}$	$\delta^{18}O_{p2}$	$\delta^{18}O_{p3}$	$\delta^{18}O_{p4}$	$\delta^{18}O_{p5}$	$\delta^{18}O_{p6}$	$\delta^{18}O_{p7}$	$\delta^{18}O_{p8}$	$\delta^{18}O_{p9}$	$\delta^{18}O_{p10}$	$\delta^{18}O_{p11}$	$\delta^{18}O_{p12}$
$\delta^{18}O_{p1}$	1.000											
$\delta^{18}O_{p2}$	0.805	1.000										
$\delta^{18}O_{p3}$	0.461	0.775	1.000									
$\delta^{18}O_{p4}$	-0.437	-0.194	0.290	1.000								
$\delta^{18}O_{p5}$	-0.641	-0.661	-0.365	**0.690**	1.000							
$\delta^{18}O_{p6}$	-0.222	-0.497	-0.573	0.117	**0.674**	1.000						
$\delta^{18}O_{p7}$	0.528	0.239	-0.110	-0.311	-0.046	**0.552**	1.000					
$\delta^{18}O_{p8}$	0.557	0.595	0.526	-0.093	-0.300	0.036	**0.548**	1.000				
$\delta^{18}O_{p9}$	0.585	0.640	0.592	-0.123	-0.444	-0.164	0.378	**0.855**	1.000			
$\delta^{18}O_{p10}$	0.635	0.720	0.642	-0.139	-0.528	-0.310	0.372	0.715	0.876	1.000		
$\delta^{18}O_{p11}$	0.854	0.745	0.403	-0.452	-0.664	-0.213	0.577	0.502	0.552	0.684	1.000	
$\delta^{18}O_{p12}$	0.867	0.729	0.337	-0.458	-0.583	-0.102	0.618	0.518	0.519	0.583	0.915	1.000

Note S14. Correlations of August-September and April-May EA $\delta^{18}O_p$ with the EA $\delta^{18}O_v$

The EA August-September and April-May $\delta^{18}O_p$ series are closely related to the concurrent local atmospheric water vapor $\delta^{18}O_v$ (Table S10). Strongest positive correlations exist between the August-September (April-May) $\delta^{18}O_p$ and the $\delta^{18}O_v$ at 600-700 hPa (850-925 hPa) in the mid-lower troposphere (Table S10). For example, the correlation coefficient of August-September (April-May) $\delta^{18}O_p$ with the 700-hPa $\delta^{18}O_v$ is 0.902 (0.901), as seen in Fig. S8. Similarly, the correlations of $\delta^{18}O_p$ with the whole-troposphere $\delta^{18}O_v$ are also very strong, r=0.893 for August-September and r=0.953 for April-May (Table S10).

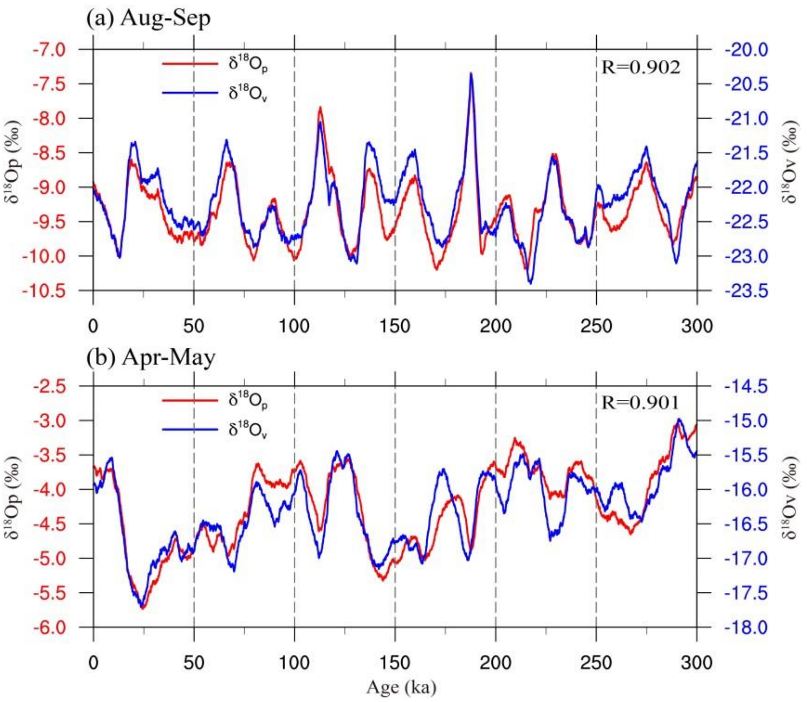

Fig. S8 Comparisons of the August-September (a) and April-May (b) EA precipitation $\delta^{18}O_p$ and 700-hPa water vapor $\delta^{18}O_v$ series.

Table S10 Correlation coefficients between the August-September (the first row) or April-May (the second row) EA precipitation $\delta^{18}O_p$ and water vapor $\delta^{18}O_v$ at different isobaric levels, as well as for the whole troposphere (300hPa-surface).

	200hPa	300hPa	400hPa	500hPa	600hPa	700hPa	850hPa	925hPa	300hPa-Surface
Aug-Sep $\delta^{18}O_p \sim \delta^{18}O_v$	0.159	0.414	0.585	0.750	0.926	0.902	0.698	0.517	0.893
Apr-May $\delta^{18}O_p \sim \delta^{18}O_v$	0.094	0.200	0.366	0.680	0.819	0.901	0.977	0.967	0.953

Note S15. Correlations of the insolation, GIV and GHGs with the August-September and April-May EA $\delta^{18}O_p$ and water vapor content

In order to establish the possible association of the EA $\delta^{18}O_p$ variation with the orbital forcing, we calculated the correlations of EA August-September and April-May $\delta^{18}O_p$, which contribute most to the EA annual $\delta^{18}O_p$ changes, with the NH 30°N insolation reflecting the orbital forcing. The result shows that there are significant negative correlations between the August-September $\delta^{18}O_p$ and July or August 30°N insolation series (r=−0.691 or −0.577), but the correlations between April-May $\delta^{18}O_p$ and the insolation in individual months are relatively weak (Table S11). Again, the strong positive correlations with the winter-month insolation are ignored due to their low contributions to the annual $\delta^{18}O_p$ values. Additionally, the correlation coefficient between the August-September (April-May) $\delta^{18}O_p$ and July-August (March-April) average insolation is −0.690 (0.035) (Table S12). On the other hand, the April-May $\delta^{18}O_p$ is strongly correlated with the variations of the GIV and atmospheric GHG concentrations, while August-September $\delta^{18}O_p$ has much weaker relations with these forcing factors (Table S12). Meanwhile, the August-September whole-troposphere water vapor content averaged for the EA shows strong positive correlations with the insolation during July and August, while the April-May water vapor content has strong positive correlations with March and April insolation (Table S11). These results suggest that the EA August-September and April-May $\delta^{18}O_p$ are likely controlled by the insolation and GIV (possibly including the GHG concentrations as well), respectively, due their modulating effects through regulating the large-scale atmospheric circulation patterns.

Table S11 Correlation coefficients of the January-December monthly 30 °N insolation series (S_1, S_2…S_{12}) with the EA April-May $\delta^{18}O_p$ ($\delta^{18}O_{p4\_5}$, the first row), whole-troposphere April-May water vapor content (Q_{4_5}, the second row), August-September $\delta^{18}O_p$ ($\delta^{18}O_{p8\_9}$, the third row), and whole-troposphere August-September water vapor content (Q_{8_9}, the fourth row)

	S_1	S_2	S_3	S_4	S_5	S_6	S_7	S_8	S_9	S_{10}	S_{11}	S_{12}
$\delta^{18}O_{p4\_5}$	-0.324	-0.223	-0.043	0.089	0.185	0.243	0.222	0.081	-0.086	-0.191	-0.254	-0.302
Q_{4_5}	-0.076	0.491	0.868	0.917	0.741	0.335	-0.285	-0.826	-0.875	-0.643	-0.400	-0.267
$\delta^{18}O_{p8\_9}$	0.743	0.761	0.499	0.188	-0.137	-0.466	-0.691	-0.572	-0.161	0.221	0.486	0.637
Q_{8_9}	-0.558	-0.871	-0.823	-0.554	-0.167	0.317	0.774	0.857	0.478	0.039	-0.265	-0.399

Table S12 Correlation coefficients of the April-May ($\delta^{18}O_{p4\_5}$, the first row) and August-September ($\delta^{18}O_{p8\_9}$, the second row) $\delta^{18}O_p$ in the EA region with the 30 °N insolation series in March-April (S_{3_4}) and July-August (S_{7_8}), GIV, and GHGs.

	S_{3_4}	S_{7_8}	GIV	GHGs
$\delta^{18}O_{p4\_5}$	0.035	0.167	-0.797	0.667
$\delta^{18}O_{p8\_9}$	0.329	-0.690	0.289	-0.087

References

1. Kutzbach J, Liu XD and Liu ZY *et al*. Simulation of the evolutionary response of global summer monsoons to orbital forcing over the past 280,000 years. *Clim Dyn* 2008; **30**: 567–579.
2. Xie XX, Liu XD and Chen GS *et al*. A transient modeling study of the latitude dependence of East Asian winter monsoon variations on orbital timescales. *Geophys Res Lett* 2019; **46**: 7565–7573.
3. Berger A. Long-term variations of daily insolation and Quaternary climatic changes. *J Atmos Sci* 1978; **35**: 2362–2367.
4. Petit JR, Jouzel J and Raynaud D *et al*. Climate and atmospheric history of the past 420,000 years from the Vostok ice core, Antarctica. *Nature* 1999; **399**: 429–436.
5. Lisiecki LE and Raymo ME. A Pliocene-Pleistocene stack of 57 globally distributed benthic $\delta^{18}O$ records. *Paleoceanography* 2005; **20**: PA1003.

6. Peltier WR. Global glacial isostasy and the surface of the ice-age Earth: the ICE-5G (VM2) model and GRACE. *Annu Rev Earth Planet Sci* 2004; **32**: 111–149.
7. De Boer B, Van de Wal RSW and Lourens LJ *et al*. A continuous simulation of global ice volume over the past 1 million years with 3-D ice-sheet models. *Clim Dyn* 2013; **41**: 1365–1384.
8. Talkner P and Weber RO. Power spectrum and detrended fluctuation analysis: Application to daily temperatures. *Phys Rev E* 2000; **62**: 150.
9. Hannan EJ. Multiple time series. Wiley, New York, 1970, 536
10. Bretherton CS, Smith C and Wallace JM. An Intercomparison of methods for finding coupled patterns in climate data. *J Clim* 1992. **5**: 541–560.
11. Von Storch H, Zwiers FW. Statistical Analysis in Climate Research. Cambridge University Press: Cambridge, UK, 2002.
12. DelSole T and Tippett MK. Statistical methods for climate scientists. Cambridge University Press, 2021.
13. Montgomery DC, Peck EA and Vining GG. Introduction to linear regression analysis, 5th ed. (Wiley series in probability and statistics, Vol. 821), John Wiley & Sons, 2012.
14. Harris I, Osborn TJ and Jones P *et al*. Version 4 of the CRU TS monthly high-resolution gridded multivariate climate dataset. *Sci Data* 2020; **7**: 1–18.
15. Xie PP and Arkin PA. Global precipitation: A 17-year monthly analysis based on gauge observations, satellite estimates, and numerical model outputs. *Bull Am Meteorol Soc* 1997; **78**: 2539–2558.
16. Wang T, Li TY and Zhang J *et al*. A Climatological Interpretation of Precipitation $\delta^{18}O$ across Siberia and Central Asia. *Water* 2020; **12**: 2132.
17. Le Duy N, Heidbüchel I and Meyer H *et al*. What controls the stable isotope composition of precipitation in the Mekong Delta? A model-based statistical approach. Hydrol. *Earth Syst Sci* 2018; **22**: 1239–1262.
18. Cai ZY, Tian LD and Bowen GJ. Spatial-seasonal patterns reveal large-scale atmospheric controls on Asian Monsoon precipitation water isotope ratios. *Earth Planet Sci Lett* 2018; **503**: 158–169.
19. Hendy CH. The isotopic geochemistry of speleothems–I. The calculation of the effects of

different modes of formation on the isotopic composition of speleothems and their applicability as palaeoclimatic indicators. *Geochimica et Cosmochimica Acta* 1971; **35**: 801-824.

20. McDermott F. Palaeo-climate reconstruction from stable isotope variations in speleothems: a review. *Quat Sci Rev* 2004; **23**: 901–918.
21. Luo WJ and Wang SJ. Transmission of oxygen isotope signals of precipitation-soil water-drip water and its implications in Liangfeng Cave of Guizhou, China. *Chin Sci Bull* 2008; **53**: 3364-3370.
22. Cheng H, Edwards RL and Sinha A *et al*. The Asian monsoon over the past 640,000 years and ice age terminations. *Nature* 2016; **534**: 640–646.
23. Lewis SC, LeGrande AN and Kelley M *et al*. Water vapour source impacts on oxygen isotope variability in tropical precipitation during Heinrich events. *Clim Past* 2010; **6**: 325–343.
24. Pausata FS, Battisti DS and Nisancioglu KH *et al*. Chinese stalagmite $\delta^{18}O$ controlled by changes in the Indian monsoon during a simulated Heinrich event. *Nat Geosci* 2011; **4**: 474–480.
25. Battisti DS, Ding QH and Roe GH. Coherent pan-Asian climatic and isotopic response to orbital forcing of tropical insolation. *J Geophys Res Atmos* 2014; **119**: 11–997.
26. Liu ZY, Wen XY and Brady EC *et al*. Chinese cave records and the East Asia summer monsoon. *Quat Sci Rev* 2014; **83**: 115–128.
27. Ruddiman WF. Orbital insolation, ice volume, and greenhouse gases. *Quat Sci Rev* 2003, **22**: 1597–1629.
